# Genetic Aspects of Reproductive Function Disorders in Men with Impaired Semen Parameters

**DOI:** 10.3390/medsci14060601

**Published:** 2026-09-24

**Authors:** Nina Kulchenko, Galina Myandina, Hasan Alhejoj, Alexandr Strachuk, Vladislav Bolshakov, Tatiana Demura, Nina Paramonova, Oksana Kichigina, Anna Romanovskya, Marina Krot, Artemii Mingazov, Yulii Shidlovskii, Grigory Demyashkin

**Affiliations:** 1 Research and Educational Resource Center for Immunophenotyping, Digital Spatial Profiling and Ultrastructural Analysis Innovative Technologies, Peoples’ Friendship University of Russia (RUDN University), Miklukho-Maklaya St., 6, 117198 Moscow, Russia; kle-kni@mail.ru (N.K.); myandina_gi@pfur.ru (G.M.); hasan_alhejoj@yahoo.com (H.A.); dragsyar@rambler.ru (A.S.); 2 Department of Industrial Pharmacy, Institute of Professional Education, I.M. Sechenov First Moscow State Medical University (Sechenov University), Trubetskaya St., 8/2, 119048 Moscow, Russia; vmbolshakov@gmail.com; 3 Institute for Clinical Morphology and Digital Pathology, I.M. Sechenov First Moscow State Medical University (Sechenov University), Trubetskaya St., 8/2, 119048 Moscow, Russia; demura_t_a@staff.sechenov.ru (T.D.); paramonova_n_b@staff.sechenov.ru (N.P.); kichigina_o_n_1@staff.sechenov.ru (O.K.); kukleva_msmu@mail.ru (A.R.); krot_m_a@staff.sechenov.ru (M.K.); 4 University of Graz, Universitätsplatz 3, 8010 Graz, Austria; mingazovartyom1@gmail.com; 5 Department of Biology and General Genetics, Federal State Autonomous Educational Instution of Higher Education, I.M. Sechenov First Moscow State Medical University (Sechenov University), Trubetskaya St., 8/2, 119048 Moscow, Russia; shidlovskiy_yu_v@staff.sechenov.ru

**Keywords:** male infertility, spermatogenesis, asthenozoospermia, oligozoospermia, teratozoospermia, azoospermia, genes, *MTHFR*, *MTR*, *MTRR*, *CAT*, *GSTP1*, *PDE7B*, *SEPS1*

## Abstract

Background: Male infertility is a pressing clinical and social issue, affecting approximately 30–50% of infertile couples. Despite extensive research, the etiology remains unknown in a considerable proportion of cases, underscoring the need for molecular genetic investigations into spermatogenesis disorders. Methods: the study enrolled 187 European men (68 fertile, 119 infertile). Infertile patients were stratified into four subgroups: azoospermia (*n* = 28), asthenozoospermia (*n* = 32), teratozoospermia (*n* = 25), and oligozoospermia (*n* = 34). Genotyping was performed for polymorphisms in folate metabolism (*MTHFR* C677T, *MTHFR* A1298C, *MTR* A2756G, *MTRR* A66G), redox system (*CAT*-262C > T, *GSTP1* Ile105Val, *GSTP1* Ala114Val), phosphodiesterase (*PDE7B* G/A), and selenoprotein (*SEPS1* G-105A) genes. Statistical analyses included χ^2^ tests, logistic regression, genetic risk score (GRS) calculation, and ROC analysis, with Benjamini–Hochberg correction for multiple comparisons (FDR, q < 0.05 considered significant). Groups were comparable in age, BMI, and hormonal profiles. Results: in oligozoospermia, nominal associations were detected with *MTR* A2756G (OR = 2.70; *p* = 0.003), *CAT*-262T (OR = 3.12; *p* = 0.0005), and *SEPS1* G-105A (OR = 3.45; *p* = 0.0001); after FDR correction, only *CAT* and *SEPS1* remained significant. Multivariate regression, adjusting for age, BMI, and hormones, confirmed independent associations for *CAT* (aOR = 2.34; *p* = 0.002; q = 0.04) and *SEPS1* (aOR = 2.89; *p* = 0.0001; q = 0.002). *MTR* A2756G showed nominal associations with astheno-, terato-, and oligozoospermia, but none survived FDR correction. *CAT*-262T was associated with oligozoospermia (q = 0.04) and borderline with asthenozoospermia (q = 0.08); *SEPS1* was significantly associated with both asthenozoospermia (q = 0.04) and oligozoospermia (q = 0.004). GRS was highest in oligozoospermia (3.4 ± 1.5) and significantly elevated in asthenozoospermia and teratozoospermia (3.1 ± 1.2 and 3.2 ± 1.4, respectively), but not in azoospermia (2.3 ± 1.1; *p* = 0.087). GRS ≥ 3 was found in 64.7% of infertile men vs. 26.5% of fertile controls, corresponding to a 5-fold increased risk (χ^2^ = 22.4; *p* < 0.001). The combined GRS achieved an AUC of 0.74 (95% CI 0.67–0.81), with 68.2% sensitivity and 76.5% specificity at a cut-off of ≥3. No associations with azoospermia reached significance for any polymorphism; this subgroup had limited power (*n* = 28). Conclusions: in this exploratory study, *CAT* and *SEPS1* polymorphisms demonstrated FDR-significant associations with oligozoospermia and asthenozoospermia in European men. The cumulative genetic score (AUC = 0.74) may serve as a useful tool for risk stratification, but these findings require replication in larger independent cohorts before clinical translation.

## 1. Introduction

Infertility is one of the most important problems in medicine, demography, and economics. Clinical infertility is defined as the inability of a couple to conceive after 12 months of unprotected intercourse. The male factor accounts for 30–50% of infertility cases [1,2,3,4,5,6], which is largely attributed to a steady decline in quantitative and qualitative sperm parameters over recent years [7,8,9].

The spectrum of sperm abnormalities in men with infertility varies from a decrease in sperm count (oligozoospermia), to reduced motility (asthenozoospermia), abnormal morphology (teratozoospermia), and complete absence of spermatozoa (azoospermia) [10,11]. Despite extensive research on these spermiological syndromes (by both andrologists and morphologists), the role of genetic factors in their etiopathogenesis remains poorly understood [12].

In this context, it is important to distinguish key components of genetic regulation: in addition to chromosomal abnormalities and well-established mutations, reduced expression or functional deficiency of proteins that regulate normal spermatogenesis is equally significant. Such proteins are responsible for sperm motility, as well as cell adhesion during division and maturation [13].

To date, several specific molecular defects disrupting these processes have been well characterized. Currently proven genetic causes of male infertility associated with impaired semen parameters include Klinefelter syndrome, chromosomal aberrations, Y-chromosome microdeletions in the AZF locus, and mutations in the *CFTR* and *AR* genes [14,15,16,17,18,19]. However, the etiology of other cases remains unclear. It is assumed that in idiopathic cases, a combination of environmental factors and polymorphisms of many genes affects the spermatogenic epithelium [20].

It is known that more than 2000 human genes are involved in the regulation of intratesticular homeostasis, proliferation and differentiation of spermatogenic cells. Primarily, these are genes whose products participate in cellular metabolism and protective mechanisms [21,22].

Variations in genes of folate metabolism (*MTHFR*, *MTR*, *MTRR*), phosphodiesterase (*PDE7B*), selenoproteins (*SEPS1*), and antioxidant systems (*CAT*, *GSTP1*) can impair enzymatic activity and neutralization of reactive oxygen species [23,24,25,26]. This leads to elevated cytokine levels and germ cell damage [27]. The folate cycle regulates DNA methylation, which is critical during spermatogonial divisions; its impairment leads to loss of epigenetic information and impaired semen parameters [28]. The redox system protects spermatozoa from oxidative damage; its deficiency elevates DNA fragmentation, reduces motility, and causes morphological abnormalities [29]. Selenoproteins such as *SEPS1* provide antioxidant protection [30]. Therefore, analyzing associations of these polymorphisms with spermatogenic disorders is a promising direction.

Despite significant advances in assisted reproductive technologies, 3–4% of couples remain childless [31]. Therefore, there is a compelling need for molecular genetic studies aimed at elucidating the pathogenic mechanisms underlying impaired sperm production of various etiologies. In this regard, it is highly relevant to investigate the expression and polymorphisms of genes responsible for antioxidant defense and cellular protection against oxidative stress, given its critical role in impairing spermatogenesis and male reproductive function.

## 2. Research Objective

Aim of the study: To evaluate the association of genetic polymorphisms regulating folate metabolism, the redox system, phosphodiesterase, and selenoprotein with the risk of developing impaired semen parameters.

Research objectives:

- To analyze the distribution of the following genetic polymorphisms among infertile and fertile men:
  - Folate cycle regulatory genes: *MTHFR* C677T (rs1801133) and A1298C (rs1801131), *MTRR* A66G (rs1801394), and *MTR* A2756G (rs1805087);
  - Redox system regulatory genes: *CAT*-262 C > T (rs1001179), *GSTP1* A313G (Ile105Val, rs1695), and *GSTP1* C341T (Ala114Val, rs1138272);
  - Phosphodiesterase synthesis gene: *PDE7B* G/A (rs7774640);
  - Selenoprotein S1 synthesis gene: *SEPS1 G-105A* (rs28665122).
- To determine the potential associations and predictive roles of these polymorphisms in the risk of developing specific forms of impaired semen parameters.

For the subsequent construction of the genetic risk score (GRS), only four candidate SNPs with well-documented biological relevance (*MTR* A2756G, *CAT*-262T, *GSTP1* Ala114Val, and *SEPS1* G-105A) were pre-specified based on published evidence.

## 3. Material and Methods of Research

### 3.1. Patients

The study included European men (*n* = 187), divided by fertility (*n* = 68) and infertility (*n* = 119). Depending on the specific semen abnormalities, infertile patients (*n* = 119) were divided into four subgroups: azoospermia (*n* = 28), asthenozoospermia (*n* = 32), teratozoospermia (*n* = 25), and oligozoospermia (*n* = 34).

All participants were recruited from the clinic of the RUDN University Hospital between January 2023 and December 2025. Fertile controls were recruited from men who underwent routine screenings (exhibiting normozoospermia according to the WHO 2021 guidelines and having no history of infertility) and confirmed having biological children. Participation was voluntary; no financial compensation was provided.

The main inclusion criteria for patients with infertility: age from 18 to 40 years; established fact of infertility without the use of contraception (ICD 10-N46, Male infertility); no history of bilateral testicular lesions; no testicular hypoplasia on both sides; no female factor of infertility in the partner.

The Local Ethics Committee of the RUDN University approved this study (Protocol #11 from 24 November 2024[M1] [AM2]). This article adhered to the Strengthening the Reporting of Observational Studies in Epidemiology (STROBE) guidelines. All procedures followed the principles of the Declaration of Helsinki. All participants signed a standard informed consent agreement to participate in the study. [M3] [AM4]. All participants provided written informed consent for participation in the study and for the use of their biological samples and clinical data for research purposes. All samples and data were handled in accordance with the Guidelines on Human Biobanks and Genetic Research Databases, ensuring proper collection, anonymization, processing, and storage under internationally recognized ethical and quality standards.

Exclusion criteria: combined forms of infertility (e.g., asthenoteratozoospermia); smoking; obstructive forms of infertility; endocrine factors of infertility; severe concomitant pathology at the time of examination; diseases caused by STIs (sexually transmitted infections); karyotype abnormalities; *CFTR* gene mutations and AZF locus deletions.

Prior to inclusion, all participants underwent a standardized clinical evaluation to verify the exclusion criteria. Hormonal assessment included measurement of serum follicle-stimulating hormone (FSH), luteinizing hormone (LH), testosterone, and prolactin levels to exclude endocrine causes of infertility. Karyotyping was performed in all infertile men to exclude chromosomal anomalies. Molecular genetic testing was conducted to rule out *CFTR* gene mutations and AZF locus microdeletions. Screening for STIs, including *Chlamydia trachomatis*, *Mycoplasma genitalium*, *Ureaplasma urealyticum*, and *Neisseria gonorrhoeae*, was performed using PCR-based assays. Obstructive azoospermia was excluded based on absence of obstruction at Micro-TESE; ultrasound and clinical findings were supportive.

To ensure comparability between the groups and to eliminate the influence of potential confounding factors, a comparative analysis of the baseline clinical and demographic characteristics was performed between the groups of fertile and infertile men, as well as between the patient subgroups with various forms of impaired semen parameters (Table 1).

As presented in Table 1, all studied groups were comparable in terms of age, body mass index (BMI), duration of infertility (within the infertile patient subgroups), and serum levels of FSH, LH, testosterone, and prolactin. No statistically significant differences were observed between the groups (*p* > 0.05 for all comparisons). The comparability of the groups across these parameters minimizes the impact of these potential confounding factors on the genetic analysis results, confirming that the identified associations of polymorphisms with impaired semen parameters are not driven by differences in age, BMI, or hormonal status between the groups.

### 3.2. Genetic Analysis

Genomic DNA was isolated from peripheral blood leukocytes using the “DNA-EXTRAN-1-blood” commercial kit (LLC “Sintol”, Moscow, Russia) in accordance with the manufacturer’s protocol. Venous blood samples were collected in vacuum tubes containing EDTA as an anticoagulant and stored at −20 °C prior to processing. The quantity and purity of the extracted DNA were evaluated spectrophotometrically to ensure sufficient template quality for subsequent molecular analyses. The study population comprised two main cohorts: a control group of fertile men with normozoospermia (*n* = 68) and a patient group with impaired semen parameters (*n* = 119). Two distinct genotyping methods were employed for the study participants. First, determination of the *MTHFR* C677T, *MTHFR* A1298C, *MTR* A2756G, *MTRR* A66G, *CAT*-262 C > T, *GSTP1* A313G (Ile105Val), *GSTP1* C341T (Ala114Val), and *PDE7B* G/A genotypes was performed via Real-Time PCR. The reaction mixtures were prepared using allele-specific primers and fluorescently labeled probes. Amplification and allelic discrimination were executed using a CFX96 Real-Time thermal cycler (Bio-Rad Laboratories, Inc., Hercules, CA, USA) equipped with CFX Manager software (version 3.1, Bio-Rad Laboratories, Inc., Hercules, CA, USA), which provided automated fluorescence detection and precise genotype assignment.

Further, the distribution of the G-105A polymorphism (rs28665122) of the *SEPS1* gene was investigated by end-point polymerase chain reaction (PCR) using a Tercik thermal cycler (LLC “NPO DNA-Technology”, Moscow, Russia). Amplification of the target DNA region was performed using specialized SNP-express reagent kits (CJSC “Evrogen”, Moscow, Russia). Following successful amplification, the resulting PCR products underwent site-specific enzymatic cleavage through treatment with Mox20I restriction endonuclease (“Sibenzyme”, Novosibirsk, Russia) under optimal incubation conditions. The restriction fragments were subsequently separated by agarose gel electrophoresis to ensure accurate visualization. Subsequent restriction fragment length polymorphism (RFLP) analysis was carried out to determine the individual patient genotypes and calculate allele frequencies within the study cohort.

### 3.3. Statistical Analysis

Statistical analysis was performed using Statistica 14.1.0 software, with the significance level set at *p* ≤ 0.05. Hardy–Weinberg equilibrium was checked by χ^2^ and Fisher’s exact test (all SNPs were in HWE, *p* > 0.05). Associations were estimated by OR and 95% CI. Continuous variables were compared by *t*-test or ANOVA, categorical by χ^2^.

Multivariate logistic regression was performed with adjustment for age, BMI, and hormones. Interactions (epistasis) were tested using a multiplicative model. Genetic risk score (GRS) was calculated as the sum of risk alleles for four pre-specified SNPs (*MTR* A2756G, *CAT*-262T, *GSTP1* Ala114Val, *SEPS1* G-105A) selected from the literature before data analysis. Bootstrap validation (1000 resamples) was used to assess GRS stability. ROC analysis with AUC. To correct for multiple comparisons (108 tests), we applied Benjamini–Hochberg false discovery rate (FDR) with a threshold of q < 0.05. Sample size was estimated in G*Power 3.1 (α = 0.05, two-sided). With a risk-allele carrier frequency of 17–28% in controls and a case:control ratio of 119:68, 230 participants are required to detect OR = 2.5 at 80% power, and 154 participants to detect OR = 3.0. The primary endpoint was the comparison of the whole infertile group (*n* = 119) with fertile controls (*n* = 68). For this comparison the study was adequately powered for *SEPS1* G-105A (92%) and approached adequate power for *CAT*-262T (78% for carrier status), the two loci that remained significant after FDR correction. Power in the subgroups was lower, and all subgroup comparisons were therefore treated as exploratory. Comparisons were divided into primary (univariate and multivariate analyses in the whole infertile group) and exploratory (subgroup analyses and gene–gene interaction tests). The same division is used throughout Section 4. Post hoc power was calculated in G*Power 3.1 and is reported for each subgroup in Table 2. Results are reported as nominal *p*-values; those significant after FDR are explicitly indicated.

## 4. Results

### 4.1. Analysis of the Frequency of Polymorphisms of Folate Cycle Genes in Patients with Impaired Sperm

The Results of allele and genotype distribution for *MTHFR* C677T, *MTHFR* A1298C, *MTR* A2756G, and *MTRR* A66G polymorphisms among fertile and infertile men are presented in Table 3.

The analysis of observed and expected frequencies showed that, among infertile men with impaired semen parameters, the distribution of genotype frequencies for the *MTHFR* C677T, *MTHFR* A1298C and *MTRR* A66G polymorphisms did not differ statistically from that in the group of fertile men (*p* > 0.05). However, the distribution of genotypes for the *MTR* A2756G polymorphism differed statistically significantly between infertile and fertile men (*p* = 0.012). A comparative analysis of allelic variants revealed a statistically significant difference in the G allele at the *MTR* A2756G locus (χ^2^ = 7.892; OR = 2.12; 95% CI 1.27–3.54; *p* = 0.005), whilst the allele frequencies for *MTHFR* C677T, *MTHFR* A1298C and *MTRR* A66G did not differ significantly (*p* > 0.05). It should be noted, however, that after applying the Benjamini–Hochberg correction for multiple testing, none of these associations remained statistically significant (q > 0.05 for all comparisons). The results are shown in Figure 1 and Figure 2.

Then, data on the distribution of genotypes for polymorphisms C677T (rs1801133) and A1298C (rs1801131) of the *MTHFR* gene, A66G of the *MTRR* gene (rs1801394) and A2756G of the *MTR* gene (rs1805087) were compared with subgroups of infertile men (Table 4).

When assessing the frequency of distribution of genotypes and alleles of the C677T *MTHFR* gene and A66G *MTRR* gene loci, they do not statistically differ in the control group and in the subgroups of infertile men with different clinical forms of impaired semen parameters (*p* > 0.05). At the same time, no statistically significant difference was found in the allele frequencies for the A66G polymorphism of the *MTRR* gene between the subgroup of men with teratozoospermia and the group of fertile men (χ^2^ = 2.95; OR = 1.78; *p* = 0.086). Also, we did not detect associations between the C677T *MTHFR*, A1298C *MTHFR*, A2756G *MTR*, A66G *MTRR* loci and the risk of infertility in the subgroup of men with azoospermia (*p* > 0.05). In the oligozoospermia subgroup, nominal associations were observed for the *MTHFR* C677T T allele (χ^2^ = 4.89; OR = 1.67; *p* = 0.027), *MTHFR* A1298C C allele (χ^2^ = 4.12; OR = 1.81; *p* = 0.042), and *MTR* A2756G G allele (χ^2^ = 9.02; OR = 2.70; 95% CI 1.42–5.14; *p* = 0.003) when compared with fertile men (Table 4).

Among patients with asthenozoospermia, the distribution of the A1298C *MTHFR* and A2756G *MTR* genotypes was statistically significantly different from the control group (χ^2^ = 8.12; *p* = 0.017) and (χ^2^ = 8.00; *p* = 0.018), respectively (Figure 3). Among patients with asthenozoospermia, the frequency of carriers of minor alleles for the A1298C polymorphism of the *MTHFR* gene (genotypes AC + CC) and A2756G of the *MTR* gene (genotypes AG + GG) was statistically significantly higher than in the control group (χ^2^ = 5.80, OR = 3.17, 95% CI 1.21–8.32; *p* = 0.016) and (χ^2^ = 4.69; OR = 2.63; 95% CI 1.08–6.37; *p* = 0.030), respectively. It should be noted, however, that after applying the Benjamini–Hochberg correction for multiple testing, none of these associations remained statistically significant (q > 0.05 for all comparisons).

Among patients with teratozoospermia, the revealed distribution of genotype frequencies for the A2756G polymorphism of the *MTR* gene does not statistically significantly differ from the distribution of genotype frequencies among fertile men (*p* > 0.05). However, the frequencies of heterozygous and homozygous carriers of the minor allele 2756G of the *MTR* gene (genotypes AG + GG) statistically significantly differ from the control group (χ^2^ = 5.12; OR = 3.07; 95% CI 1.13–8.30; *p* = 0.024). Statistically significant differences in the frequencies of the minor allele 2756G of the *MTR* gene were also revealed in the subgroup of patients with teratozoospermia and in the control group of fertile men (χ^2^ = 4.44; OR = 2.08; 95% CI 1.05–4.14; *p* = 0.035). In the oligozoospermia subgroup, a similar association was observed for the *MTR* A2756G polymorphism: carriers of the AG + GG genotypes were more frequent compared with fertile men (χ^2^ = 7.12; OR = 3.36; 95% CI 1.38–8.19; *p* = 0.008), and the G allele frequency was also significantly higher (χ^2^ = 9.02; OR = 2.70; 95% CI 1.42–5.14; *p* = 0.003) (Table 4).

In the subgroup of patients with teratozoospermia, the distribution of genotype frequencies for the A66G polymorphism of the *MTRR* gene is not statistically different from the distribution of genotype frequencies in the control group (*p* = 0.23). Similarly, the frequencies of the minor allele 66G of the *MTRR* gene among patients with teratozoospermia did not statistically significantly differ from the control group (χ^2^ = 2.95; OR = 1.78; *p* = 0.086) (Figure 4). In the oligozoospermia subgroup, no significant differences were found for the *MTRR* A66G polymorphism (*p* > 0.05 for all comparisons). It should be noted, however, that after applying the Benjamini–Hochberg correction for multiple testing, none of the aforementioned associations for *MTR* and *MTRR* remained statistically significant (q > 0.05 for all).

Abstract 1: Although the MTR and MTHFR polymorphisms showed nominal associations with certain disorders in sperm, none of them were significant after the FDR correction.

The A2756G polymorphism of the *MTR* gene (rs1805087) was found to be nominally associated with the risk of teratozoospermia, asthenozoospermia, and oligozoospermia, while the A1298C polymorphism of the *MTHFR* gene (rs1801131) was associated with the risk of asthenozoospermia and oligozoospermia in nominal analyses. However, after correction for multiple testing (FDR), none of these associations remained statistically significant (q > 0.05).

### 4.2. Analysis of the Distribution of Polymorphisms of Genes Encoding Antioxidant Enzymes (Redox System) (CAT, GSTP1) and the Phosphodiesterase Gene (PDE7B) in Patients with Impaired Semen Parameters

The results of the analysis of the distribution of genotypes and alleles for the polymorphisms *CAT* (C262T; rs1001179), *GSTP1* (Ile/Val) (A313G; rs1695), *GSTP1* (Ala/Val) (C341T; rs1138272), and *PDE7B* (G/A; rs7774640) in infertile men with impaired semen parameters and in the group of fertile men are shown in Table 5 and Figure 5 and Figure 6.

Analysis of the results presented in Table 5 revealed that among the studied polymorphisms of antioxidant system genes (*CAT*-262C > T, *GSTP1* Ile105Val, *GSTP1* Ala114Val) and the phosphodiesterase gene (*PDE7B* G/A), only the *CAT*-262C > T polymorphism demonstrated statistically significant differences between the overall group of infertile men (*n* = 119) and fertile controls (*n* = 68), and these differences remained significant after stringent correction for multiple comparisons (FDR). In particular, the homozygous CC genotype was less frequent among infertile men (47% vs. 69% in fertile men), whereas the frequency of CT heterozygotes (42% vs. 28%) and TT homozygotes (11% vs. 3%) was higher. Carriage of the minor T allele in any copy number (genotypes CT + TT) was associated with an increased risk of impaired semen parameters: the odds ratio was 2.51 (95% CI 1.32–4.78) with *p* = 0.004, and the adjusted q = 0.04 confirmed the significance of this association. A similar trend was observed at the allelic level: the T-allele frequency in infertile men was nearly twice as high (32% vs. 17%), corresponding to OR = 2.31 (95% CI 1.32–4.04, *p* = 0.005), with a q-value at the border of statistical significance (q = 0.05), suggesting that this allele may be a potential risk factor warranting further investigation.

In contrast to *CAT*, the *GSTP1* polymorphisms did not show significant associations after correction for multiple comparisons. For the Ile105Val locus (rs1695), the frequency of the minor G allele was slightly higher in infertile men (37% vs. 30%), but this difference did not reach nominal significance (*p* = 0.181, q = 0.85), and genotype distributions (AA, AG, GG) were comparable between the groups. A somewhat more pronounced, yet still statistically unsupported, trend was observed for the Ala114Val polymorphism (rs1138272): carriage of the T allele (genotypes CT + TT) was present in 34% of infertile men vs. 16% of fertile controls (OR = 2.68, *p* = 0.010), and the T-allele frequency was 18% vs. 8% (OR = 2.55, *p* = 0.009). However, after FDR correction, both associations lost significance (q = 0.10 and q = 0.09, respectively), indicating a high probability of chance findings. The *GSTP1* Ile105Val polymorphism showed no even nominal differences in the frequency of risk genotype carriage (AG + GG): 58% vs. 51%, *p* = 0.359.

Regarding the phosphodiesterase gene *PDE7B* (rs7774640), all analyses genetic models (genotypes GG, GA, AA, carriage of the A allele, and A-allele frequency) showed no significant differences between the groups: *p*-values ranged from 0.147 to 0.512, and q-values in all cases exceeded 0.85. Thus, variations in this gene do not appear to contribute to susceptibility to spermatogenic disorders in the studied European population.

For a more detailed analysis, we evaluated the distribution of genotypes and allelic variants of the *CAT*-262C > T, *GSTP1* Ile105Val, *GSTP1* Ala114Val, and *PDE7B* G > A polymorphism separately in each clinical subgroup of infertile men: patients with asthenozoospermia, teratozoospermia, oligozoospermia, and azoospermia. The corresponding data are presented in Table 6.

Stratification by clinical subtypes revealed that the strongest and most consistent associations with the *CAT*-262C > T polymorphism were confined to asthenozoospermia and oligozoospermia. In men with asthenozoospermia carriage of the minor T allele (CT + TT) was 53% vs. 30% in fertile controls (χ^2^ = 4.57; *p* = 0.033), and the T-allele frequency was 34% vs. 17% (χ^2^ = 7.61; *p* = 0.006). In oligozoospermia, these differences were even more pronounced: CT + TT in 65% (χ^2^ = 11.23; *p* = 0.0008) and T-allele in 38% (χ^2^ = 12.01; *p* = 0.0005). After FDR correction, both associations remained significant for oligozoospermia (q = 0.04 for both genotype carriage and allele frequency), whereas for asthenozoospermia they were borderline (q = 0.08 for the T-allele). In teratozoospermia, the T-allele frequency (34% vs. 17%, χ^2^ = 6.32; *p* = 0.012) and CT + TT carriage (60% vs. 30%, χ^2^ = 6.53; *p* = 0.011) were also increased, but these lost significances after FDR correction (q > 0.05). For azoospermia, all *CAT*-related parameters were similar to controls (CT + TT: 36%, *p* = 0.65; T-allele: 20%, *p* = 0.67), indicating no association with this condition.

For the *GSTP1* Ile105Val (rs1695) polymorphism, the most pronounced differences were observed in teratozoospermia: the minor G-allele frequency reached 52% vs. 30% in controls (χ^2^ = 7.58; *p* = 0.006), and AG + GG carriage was 80% vs. 51% (χ^2^ = 6.16; *p* = 0.013) (Figure 7). In oligozoospermia, only a trend toward higher G-allele frequency was seen (43% vs. 30%, χ^2^ = 4.12; *p* = 0.042), which did not survive FDR correction (q = 0.42). No significant differences were found for asthenozoospermia or azoospermia (*p* > 0.05 for all comparisons).

Regarding the *GSTP1* Ala114Val (rs1138272) polymorphism, carriage of the T allele (genotypes CT + TT) was increased in all subgroups except azoospermia: in asthenozoospermia—41% vs. 16% (χ^2^ = 7.13; *p* = 0.008), in teratozoospermia—36% vs. 16% (χ^2^ = 4.26; *p* = 0.039), and in oligozoospermia—38% vs. 16% (χ^2^ = 6.12; *p* = 0.013) (Figure 8). The T-allele frequency was also higher: in asthenozoospermia—22% vs. 8% (χ^2^ = 7.56; *p* = 0.006), in teratozoospermia—20% vs. 8% (χ^2^ = 5.18; *p* = 0.023), and in oligozoospermia—21% vs. 8% (χ^2^ = 6.45; *p* = 0.011). However, after correction for multiple comparisons, all these associations lost statistical significance (q > 0.05). No differences were found for azoospermia (*p* > 0.05).

The *PDE7B* G > A polymorphism showed no significant associations in any of the infertile men subgroups, with all *p*-values exceeding 0.05 and q-values above 0.8 (Table 5). This indicates that this locus does not contribute to the susceptibility to the studied forms of impaired spermatogenesis.

Abstract 2: Of the analyzed antioxidant and phosphodiesterase gene polymorphisms, only *CAT*-262C > T retained statistically significant associations after FDR correction with oligozoospermia (q = 0.04) and borderline significance with asthenozoospermia (q = 0.08), while *GSTP1* and *PDE7B* polymorphisms failed to survive multiple testing correction.

### 4.3. Analysis and Assessment of the Distribution of the Selenoprotein S1 Gene (SEPS1, G-105A, rs28665122) Polymorphism Among Infertile and Fertile Men

Analysis of genotype and allele frequency distributions of the *SEPS1* G-105A polymorphism (rs28665122) in the overall group of infertile men (*n* = 119) and fertile controls (*n* = 68) revealed highly significant differences (Table 7). The frequency of GG homozygotes was lower among infertile men (43% vs. 72% in fertile men), whereas the frequency of GA heterozygotes (51% vs. 27%) and AA homozygotes (6% vs. 1%) was higher. Carriage of the minor A allele (genotypes GA + AA) was present in 57% of infertile vs. 28% of controls, which was associated with a substantially increased risk of impaired semen parameters (OR = 3.45; 95% CI 1.82–6.54; *p* = 0.0001) (Figure 9). At the allelic level, the A-allele frequency was also significantly higher in infertile men (32% vs. 15%; OR = 2.67; 95% CI 1.58–4.51; *p* = 0.0004). After correction for multiple comparisons (FDR), all these associations remained highly statistically significant (q = 0.002 for genotype distribution, q = 0.001 for GA + AA carriage, and q = 0.004 for A-allele frequency), indicating the robustness of the findings. Thus, the *SEPS1* G-105A polymorphism is one of the strongest genetic markers associated with the risk of developing impaired semen parameters in the studied population.

To further evaluate the associations of the *SEPS1* G-105A polymorphism with specific clinical forms of impaired semen parameters, we performed a stratified analysis in subgroups of patients with asthenozoospermia, teratozoospermia, oligozoospermia, and azoospermia. The corresponding data are presented in Table 8.

Upon stratification of patients by clinical forms of impaired semen parameters, the most pronounced differences in genotype and allele distributions of the *SEPS1* G-105A polymorphism were observed in the asthenozoospermia and oligozoospermia subgroups. The overall genotype distribution (GG, GA, AA) differed significantly from controls in asthenozoospermia (χ^2^ = 10.93; *p* = 0.0042) and oligozoospermia (χ^2^ = 16.37; *p* = 0.0002), whereas for teratozoospermia the difference was less pronounced (χ^2^ = 8.14; *p* = 0.017), and for azoospermia it was not statistically significant (χ^2^ = 2.77; *p* = 0.25) (Figure 10).

In the asthenozoospermia subgroup, the frequency of the GA + AA genotypes (carriers of the minor A allele) was 62% vs. 28% in the control group, corresponding to an odds ratio of 4.30 (95% CI 1.76–10.47; *p* = 0.00095). The frequency of the A allele in this subgroup was 33% vs. 15% in controls (OR = 2.79; 95% CI 2.10–3.48; *p* = 0.0028). Even more pronounced differences were found in oligozoospermia: GA + AA carriers were present in 68% of cases vs. 28% (OR = 5.12; 95% CI 2.10–12.48; *p* = 0.0003), and the A-allele frequency reached 43% vs. 15% (OR = 3.45; 95% CI 2.11–5.63; *p* = 0.0001). In the teratozoospermia subgroup, the frequency of GA + AA was 60% (OR = 3.86; 95% CI 2.90–4.82; *p* = 0.0044), and the A-allele frequency was 32% (OR = 2.60; 95% CI 1.79–3.35; *p* = 0.0081). For azoospermia, no differences were found in GA + AA frequency (43% vs. 28%; *p* = 0.15) or in A-allele frequency (21% vs. 15%; *p* = 0.256).

After correction for multiple comparisons (FDR), the associations of *SEPS1* with asthenozoospermia (q = 0.04 for both GA + AA and the A allele) and with oligozoospermia (q = 0.004 for GA + AA and q = 0.002 for the A allele) remained statistically significant. For teratozoospermia, significance was lost (q = 0.06 and 0.08, respectively), and for azoospermia no associations were detected (q > 0.05).

Abstract 3: The *SEPS1* G-105A polymorphism showed statistically significant associations after FDR correction with asthenozoospermia (q = 0.04) and oligozoospermia (q = 0.004).

### 4.4. Comprehensive Analysis of Associations Between Genetic Polymorphisms and the Risk of Developing Pathospermia

To assess the independent contribution of genetic polymorphisms to the risk of developing pathospermia, taking into account potential confounding factors, a multivariate logistic regression analysis was performed (Table 9). The dependent variable was infertility status (presence of pathospermia), and the independent variables were genotypes of the studied polymorphisms, as well as age, BMI, and hormonal parameters (FSH, LH, testosterone, prolactin).

As shown in Table 9, after adjustment for age, BMI, and hormonal parameters, statistically significant associations with the risk of abnormal semen parameters persisted for two polymorphisms: *CAT*-262T (T allele) and *SEPS1* G-105A (A allele). For *CAT*-262T, the adjusted odds ratio (OR) was 2.34 (95% CI: 1.34–4.08; *p* = 0.002), while for *SEPS1* G-105A, it was 2.89 (95% CI: 1.78–4.69; *p* = 0.0001). Following the false discovery rate (FDR) correction for multiple comparisons, both risk factors retained their statistical significance (q = 0.04 for *CAT* and q = 0.002 for *SEPS1*), indicating the high reliability of the identified associations.

After adjustment for all factors included in the model, statistically significant associations with the risk of impaired semen parameters remained for two polymorphisms: *CAT*-262T (T allele) and *SEPS1* G-105A (A allele). For *CAT*-262T, the adjusted odds ratio was 2.34 (95% CI 1.34–4.08; *p* = 0.002), and for *SEPS1* G-105A—2.89 (95% CI 1.78–4.69; *p* = 0.0001). After applying correction for multiple comparisons (FDR), both of these factors retained statistical significance (q = 0.04 for *CAT* and q = 0.002 for *SEPS1*), indicating a high reliability of the identified associations.

The *MTR* A2756G polymorphism (G allele) showed only a nominal association in the unadjusted analysis (OR = 1.82; *p* = 0.022), which lost significance after adjustment for confounding factors and multiple comparisons (adjusted OR = 1.78; *p* = 0.031; q = 0.31). Similarly, the *GSTP1* Ala114Val polymorphism (T allele) demonstrated a nominal association in the unadjusted analysis (OR = 2.55; *p* = 0.009), but after adjustment for other factors and FDR correction, significance was not retained (adjusted OR = 2.41; *p* = 0.016; q = 0.16). The *MTHFR* A1298C polymorphism showed no significant associations in any of the analyses (*p* > 0.05 for all comparisons).

Age (adjusted OR = 1.01; *p* = 0.748) and body mass index (adjusted OR = 1.04; *p* = 0.493) did not demonstrate a statistically significant association with the risk of impaired semen parameters, which is consistent with the results of the comparative analysis of groups (Table 1) and confirms that the identified genetic associations are not driven by differences in these parameters between the groups.

In addition to the isolated effects of individual genes, gene–gene interactions (epistasis) may play an important role in shaping the predisposition to pathology, where the combination of certain alleles of different genes leads to potentiation or attenuation of their combined effect. To evaluate such interactions, we performed an analysis of pairwise combinations of polymorphisms that showed nominal associations in the univariate analysis. The results of the interaction analysis are presented in Table 10.

As shown in Table 10, no statistically significant interactions were found between individual polymorphisms (*p* > 0.05 for all pairs; q > 0.05 after FDR correction). Despite the fact that there was a nominal tendency to increase the effect when *MTR* A2756G was combined with *CAT*-262T (OR = 1.72; 95% CI 0.92–3.22; *p* = 0.091), *MTR* A2756G with *SEPS1* G-105A (OR = 1.62; 95% CI 0.92–2.86; *p* = 0.098) and *CAT*-262T with *GSTP1* Ala114Val (OR = 1.84; 95% CI 0.94–3.60; *p* = 0.073), none of these interactions reached statistical significance. The lack of statistical significance may be due to insufficient research capacity. Thus, the observed trends should be considered as exploratory and do not allow us to conclude about a synergistic effect. Nevertheless, they are biologically plausible, given the relationship between folic acid metabolism and antioxidant system genes, and deserve to be studied in larger groups.

Since individual pairwise gene–gene interactions did not reach significance, to overcome this limitation, we evaluated the cumulative additive effect of risk alleles. For this purpose, a genetic risk score (GRS) was calculated based on the identified independent risk loci, as presented in Table 11.

Analysis of Table 11 showed that in the overall group of infertile men (*n* = 119), the mean GRS was 3.1 ± 1.4, which was statistically significantly higher than in fertile men (1.8 ± 1.1; *p* < 0.001; q < 0.001). When stratified by clinical forms of impaired semen parameters, the highest GRS was observed in the oligozoospermia subgroup—3.4 ± 1.5 (median 4, interquartile range 3–5; *p* < 0.001; q < 0.001), indicating the greatest cumulative burden of risk alleles in patients with reduced sperm count. In the asthenozoospermia and teratozoospermia subgroups, GRS values were somewhat lower but still significantly exceeded the control: 3.1 ± 1.2 (*p* < 0.001; q < 0.001) and 3.2 ± 1.4 (*p* < 0.001; q < 0.001), respectively.

In contrast to the other subgroups, patients with azoospermia had a mean GRS (2.3 ± 1.1) that did not reach statistical significance compared to fertile men (*p* = 0.087; q = 0.087), which is consistent with the absence of associations for individual polymorphisms with this form of impairment. This suggests that the studied genetic variants predominantly affect qualitative and quantitative characteristics of mature spermatozoa, but not the early stages of spermatogenesis, whose disruption is characteristic of azoospermia.

Notably, the frequency of GRS ≥ 3 was 64.7% among all infertile men and only 26.5% among fertile controls (χ^2^ = 22.4; *p* < 0.001), corresponding to a 5-fold increased risk of developing impaired semen parameters. Thus, the cumulative effect of multiple risk alleles significantly exceeds the contribution of each individual polymorphism, indicating an additive nature of the influence of the studied genetic variants and confirming the feasibility of using combined genetic panels for assessing predisposition to male infertility.

To assess the diagnostic significance of the identified associations, ROC analysis was performed, and the results are presented in Table 12. For each of the polymorphisms that showed nominal associations with the risk of impaired semen parameters, the area under the ROC curve (AUC), sensitivity, and specificity were calculated, and a cut-off level for binary classification was determined. The diagnostic value of the combined measure—the genetic risk score (GRS), calculated as the sum of risk alleles for four polymorphisms (*MTR* A2756G, *CAT*-262T, *GSTP1* Ala114Val, and *SEPS1* G-105A)—was also evaluated separately.

Among the individual polymorphisms, the highest diagnostic accuracy was demonstrated by *SEPS1* G-105A: AUC was 0.66 (95% CI 0.57–0.75), sensitivity—57.6%, and specificity—72.1% at the cut-off “presence of the A allele”. For *CAT*-262T, AUC was 0.63 (95% CI 0.54–0.72), sensitivity—52.9%, and specificity—73.5%. The *MTR* A2756G and *GSTP1* Ala114Val polymorphisms showed lower diagnostic value: AUC was 0.58 (sensitivity 47.1%, specificity 70.6%) and 0.57 (sensitivity 30.6%, specificity 83.8%), respectively. Notably, *GSTP1* Ala114Val exhibited high specificity, which may indicate its potential role as a risk exclusion marker; however, its low sensitivity limits its clinical applicability.

The combination of all four polymorphisms in the form of GRS demonstrated substantially higher diagnostic accuracy compared to individual markers: AUC was 0.74 (95% CI 0.67–0.81), which significantly exceeded the values for each individual SNP. At a cut-off of GRS ≥ 3, sensitivity reached 68.2%, and specificity—76.5%. Thus, the use of a combined genetic score allows for improved differentiation between infertile and fertile men compared to the analysis of individual polymorphisms.

Abstract 4: The comprehensive analysis confirmed that the *CAT*-262T and *SEPS1* G-105A polymorphisms are independent risk factors for sperm parameter abnormalities, remaining significant after FDR correction. Furthermore, the cumulative effect of multiple risk alleles (GRS) peaked in oligozoospermia, and the combined genetic score demonstrated moderate diagnostic accuracy (AUC = 0.74), thereby justifying the utility of comprehensive genetic panels for risk stratification in male infertility.

## 5. Discussion

In this study, we investigated the association of polymorphisms in genes regulating folate metabolism, the redox system, phosphodiesterase, and selenoprotein with the risk of developing impaired semen parameters in men of European descent. After correction for multiple comparisons (FDR), only the *CAT*-262T and *SEPS1* G-105A polymorphisms retained a significant association with the risk of oligozoospermia and asthenozoospermia. All other associations were nominal and did not survive FDR correction, and therefore should be considered hypothesis-generating.

Among the folate cycle gene polymorphisms, only *MTR* A2756G showed nominal associations with teratozoospermia, asthenozoospermia, and oligozoospermia, whereas *MTHFR* A1298C was nominally associated with asthenozoospermia and oligozoospermia; however, none of these associations remained statistically significant after FDR correction (q > 0.05). These data are partially consistent with the meta-analysis by Ren Z.J. et al., which linked *MTR* A2756G to the risk of idiopathic male infertility in non-Asian populations [32], as well as with the meta-analysis by T. Tariq et al., confirming an increased risk for G allele carriers (OR = 1.21) and GG homozygotes (OR = 2.10) [33]. In our cohort, carriage of the G allele of the *MTR* gene was associated with an OR of 1.82 (*p* = 0.022) in unadjusted analysis, but this association did not survive FDR correction (q = 0.31).

Regarding *MTHFR* A1298C, our data diverge from the meta-analysis by F. Li et al., which showed no association of this polymorphism with male infertility [34]. The nominal association of *MTHFR* A1298C with asthenozoospermia observed in our study may be due to population specificity or chance, as it did not withstand FDR correction. The lack of association for *MTHFR* C677T is consistent with the data of Zhou-Cun A. et al. on the predominant role of this SNP in Asian populations [35]. We did not detect a statistically significant association for the *MTHFR* C677T polymorphism in our cohort, which may also be explained by the small sample size and exclusion of combined phenotypes. The study by K. Louie et al. confirmed the biological plausibility of the link between folate cycle defects and DNA methylation disturbances in oligozoospermia [36], which is in line with our findings.

The *CAT*-262T polymorphism demonstrated the strongest and most robust association, remaining significant after FDR correction for oligozoospermia (q = 0.04) and showing borderline significance for asthenozoospermia (q = 0.08). The minor T allele was significantly more frequent among infertile men in nominal analyses and was associated with the risk of asthenozoospermia and oligozoospermia. For the *GSTP1* Ile105Val and Ala114Val polymorphisms, only nominal associations were observed, predominantly with teratozoospermia, none of which survived FDR correction. Therefore, their specificity should be interpreted with caution as a possible chance finding.

These results are partially supported by the literature: a meta-analysis of 9 studies linked *GSTP1* Ile105Val to an increased risk of male infertility (OR = 1.29) [37], and a study conducted in Sichuan showed an increased risk of idiopathic male infertility in combination of *GSTT1* and *GSTP1* [38]. In a Chinese population, the *GSTP1* rs1695 polymorphism did not show a direct association with the risk of male infertility, acting only through gene–gene interactions [39]. The discrepancy with our data may be explained by the exclusion of combined phenotypes and insufficient power for subgroup analyses.

In the study by García Rodríguez et al., an increased risk of male infertility was associated with the wild-type CC genotype of the *CAT* C-262T gene [40]. In our work, we found that it is the minor T allele that acts as a significant risk factor for oligozoospermia (q = 0.04) and asthenozoospermia (q = 0.08), with an adjusted OR of 2.34. This discordance (known in genetic epidemiology as allelic flip) may be due to differences in genetic background and linkage disequilibrium between the Spanish and Moscow populations, as well as differences in clinical subgroup definitions. The study by Bach H.A. et al. showed a synergistic effect of *CAT* with other antioxidant genes [41], but in our study only non-significant epistatic trends were observed, which are exploratory. The work by Sadia K. et al. confirmed the significance of the catalase promoter polymorphism as a potential marker [42].

The *SEPS1* G-105A polymorphism demonstrated the strongest association, remaining significant after FDR correction for both asthenozoospermia (q = 0.04) and oligozoospermia (q = 0.004). This is consistent with literature data on the role of *SEPS1* in modulating the cellular response to oxidative stress [43,44,45]. However, the exact mechanism of *SEPS1* in spermatogenesis is not fully understood, and most previous studies linked it to autoimmune processes. We did not measure *SEPS1* expression or oxidative stress markers, so the mechanistic link remains speculative and requires functional validation.

Importantly, we did not detect statistically significant associations with azoospermia for any of the studied polymorphisms. This may be related to the limited statistical power of the subgroup (*n* = 28, power ~35%), which does not allow exclusion of moderate effects. The studied genes are predominantly involved in metabolic and antioxidant processes that play a role in maintaining the quality of mature spermatozoa, but their contribution to early stages of spermatogenesis, whose disruption is characteristic of azoospermia, is less likely. However, we cannot completely rule out their role in azoospermia; larger studies are needed to clarify this issue.

Multivariate logistic regression analysis confirmed independent, FDR-significant associations for *CAT*-262T (adjusted OR = 2.34; q = 0.04) and *SEPS1* G-105A (adjusted OR = 2.89; q = 0.002). The nominal associations of *MTR* A2756G (q = 0.31) and *GSTP1* Ala114Val (q = 0.16) did not survive FDR correction. This indicates that only *CAT* and *SEPS1* are independent risk factors for impaired semen parameters (specifically, oligozoospermia and asthenozoospermia) in our cohort. GRS analysis showed a cumulative effect: GRS was highest in the oligozoospermia subgroup (3.4 ± 1.5) and significantly elevated in asthenozoospermia and teratozoospermia, but not in azoospermia. Bootstrap validation (1000 resamples) confirmed the stability of GRS (bootstrap-derived 95% CI for AUC: 0.70–0.78). GRS ≥ 3 was observed in 64.7% of infertile and 26.5% of fertile men (χ^2^ = 22.4; *p* < 0.001), corresponding to a 5-fold risk. ROC analysis showed moderate diagnostic accuracy of GRS (AUC = 0.74; 95% CI 0.67–0.81). The GRS was constructed based on four pre-specified SNPs (selected according to literature and nominal significance in the overall group). Other SNPs that showed subgroup-specific associations may improve the GRS with a larger patient sample.

The mechanisms underlying the link between the investigated polymorphisms and pathospermia are rooted in key biological processes. Genes regulating the folate cycle (*MTHFR*, *MTR*, and *MTRR*) are responsible for the synthesis of enzymes involved in DNA methylation, particularly in rapidly dividing cells such as spermatogonia. Similarly to dietary folate deficiency, under-expression and/or defective variants of folate cycle enzymes lead to an inability to properly methylate DNA during the numerous mitotic divisions of spermatogonia; this consequently results in the loss or alteration of epigenetic information, indicating a direct correlation with semen quality impairments. In cases of a genetically determined reduction in methionine synthase (*MTR*) activity, the transfer of the methyl group from methyltetrahydrofolate to methionine—and subsequently to SAM—becomes impaired. A similar process occurs when *MTRR* activity is diminished, since the slow rejuvenation of *MTR* restricts the overall scale of methyl group transfer. Furthermore, *MTHFR* deficiency prevents the conversion of tetrahydrofolate derivatives, thereby slowing down all downstream processes. Any of these defects, either individually or in combination, acts as a rate-limiting factor: a shortage of methyl groups inhibits the DNA methylation process (Figure 11), leading to the consequences described above.

The redox system plays a substantial role in the etiology of pathospermia [46]. Its foundation is catalase, the synthesis of which is encoded by the *CAT* gene. Reactive oxygen species (ROS) cause damage to cellular components, particularly lipids, proteins, and DNA. An imbalance between ROS production and antioxidant defense proves detrimental to the functional and structural integrity of highly differentiated cells, such as spermatozoa [47]. ROS can directly damage DNA by generating oxidized adducts (e.g., 8-OHdG), leading to the formation of apurinic/apyrimidinic sites and single-strand breaks [48]. A high level of DNA fragmentation serves as a reliable indicator of male infertility. This process is also accompanied by a decrease in cell count, an increase in abnormal sperm morphologies, and, most crucially, reduced motility [49,50]. The mechanism by which the -262T allele of the *CAT* gene contributes to pathology involves insufficient catalase production, leading to damage to the sperm lipid membrane as well as the membrane structures of its organelles [51]. This triggers an energy deficit, lipid peroxidation, further membrane damage, an influx of intracellular calcium, and a cascade of reactions resulting in a significant reduction in ATP synthesis [52]. This chain of events clearly links the -262T allele of the *CAT* gene to teratozoospermia and asthenozoospermia [53].

Several limitations should be noted. First, the post-hoc analysis (Table 2) shows our study is underpowered for most subgroup comparisons, with achieved power below 80% in all subgroups; only the whole infertile group (*n* = 119) reached moderate power (64%). The minimum OR detectable at 80% power ranged from 2.73 in the whole group to 4.22 in the teratozoospermia subgroup, so the sample was sufficient only for strong genetic effects. This constraint stems from our stringent selection criteria; designed to maximize internal validity by excluding mixed phenotypes and smoking/alcohol history, they inevitably reduced the number of eligible patients. Consequently, a highly specific but small cohort of men of European descent was formed, leaving the negative findings—especially for azoospermia and several SNPs—inconclusive. Second, this study lacks data on serum folate, vitamin B12, homocysteine, and oxidative stress markers (ROS, MDA). Third, FDR correction did not confirm most nominal associations, except for *CAT*-262T and *SEPS1* G-105A. For the remaining markers, false-positive results cannot be completely ruled out, underscoring the need for replication. Fourth, the exact number of biological children per control participant was not recorded; fertility was confirmed by the documented fact of having at least one biological child together with normozoospermia according to WHO 2021 criteria. Given these constraints, the present study is exploratory and hypothesis-generating. All findings—especially those that did not survive FDR correction—require verification in larger, adequately powered, and ethnically diverse cohorts, along with functional validation, before clinical translation can be considered.

## 6. Conclusions

In this exploratory study, a comprehensive association analysis was conducted for nine polymorphisms in genes involved in folate metabolism (*MTHFR* C677T, *MTHFR* A1298C, *MTR* A2756G, *MTRR* A66G), antioxidant defense (*CAT*-262C > T, *GSTP1* Ile105Val, *GSTP1* Ala114Val), phosphodiesterase signaling (*PDE7B* G > A), and selenoprotein-mediated oxidative stress response (*SEPS1* G-105A) with the risk of various sperm parameter abnormalities. The *CAT*-262T and *SEPS1* G-105A polymorphisms were identified as independent risk factors for oligozoospermia and asthenozoospermia in European men, retaining their significance after FDR correction. The combination of four genetic markers into a genetic risk score (GRS) demonstrates moderate diagnostic accuracy (AUC = 0.74) and may be valuable for risk stratification.

Given the exploratory nature of this work and the modest sample size, these preliminary findings underscore the importance of evaluating cumulative genetic risks. The identified correlations provide a robust foundation for future hypothesis-driven research, including replication in larger independent cohorts, functional validation of the variants, and the investigation of gene–environment interactions. In the future, these polymorphisms could successfully complement a broader panel of genetic markers for individualizing approaches to male infertility diagnosis and treatment.

## Figures and Tables

**Figure 1:**
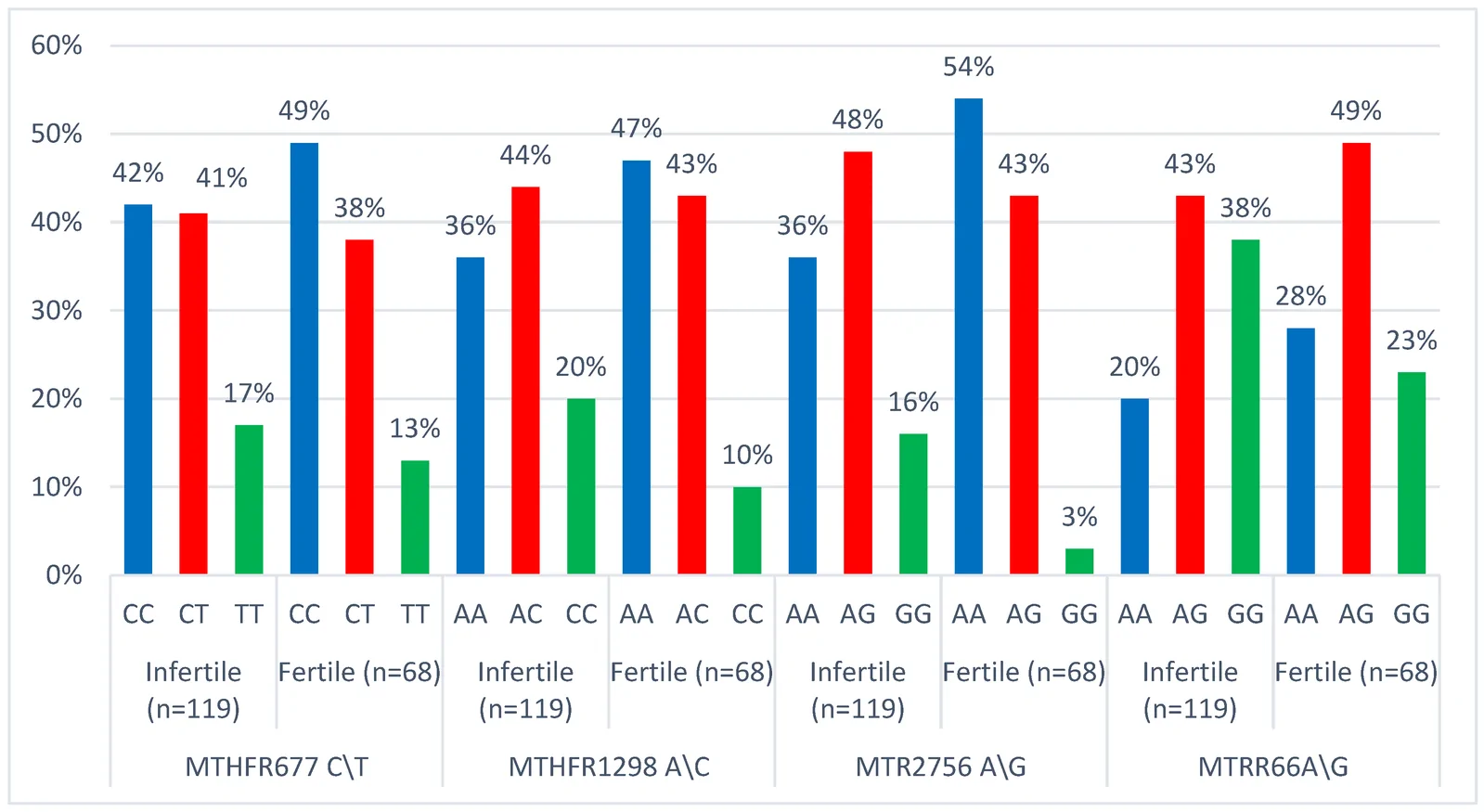
Distribution of genotype shares by polymorphisms of folate metabolism genes among fertile and infertile men.

**Figure 2:**
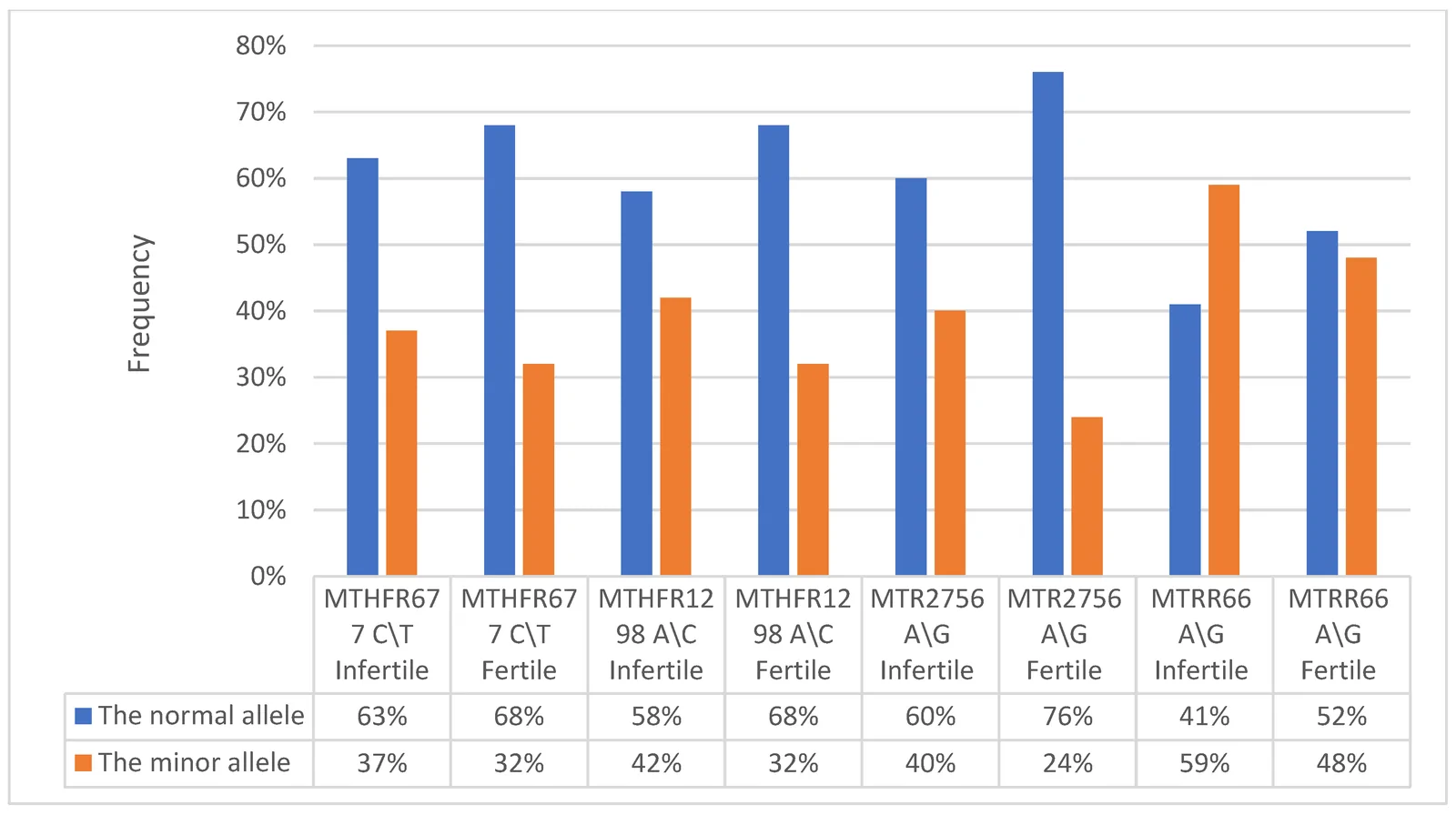
Allele frequencies of polymorphic variants C677T and A1298C of the *MTHFR* gene, A2756G of the *MTR* gene and A66G of the *MTRR* gene.

**Figure 3:**
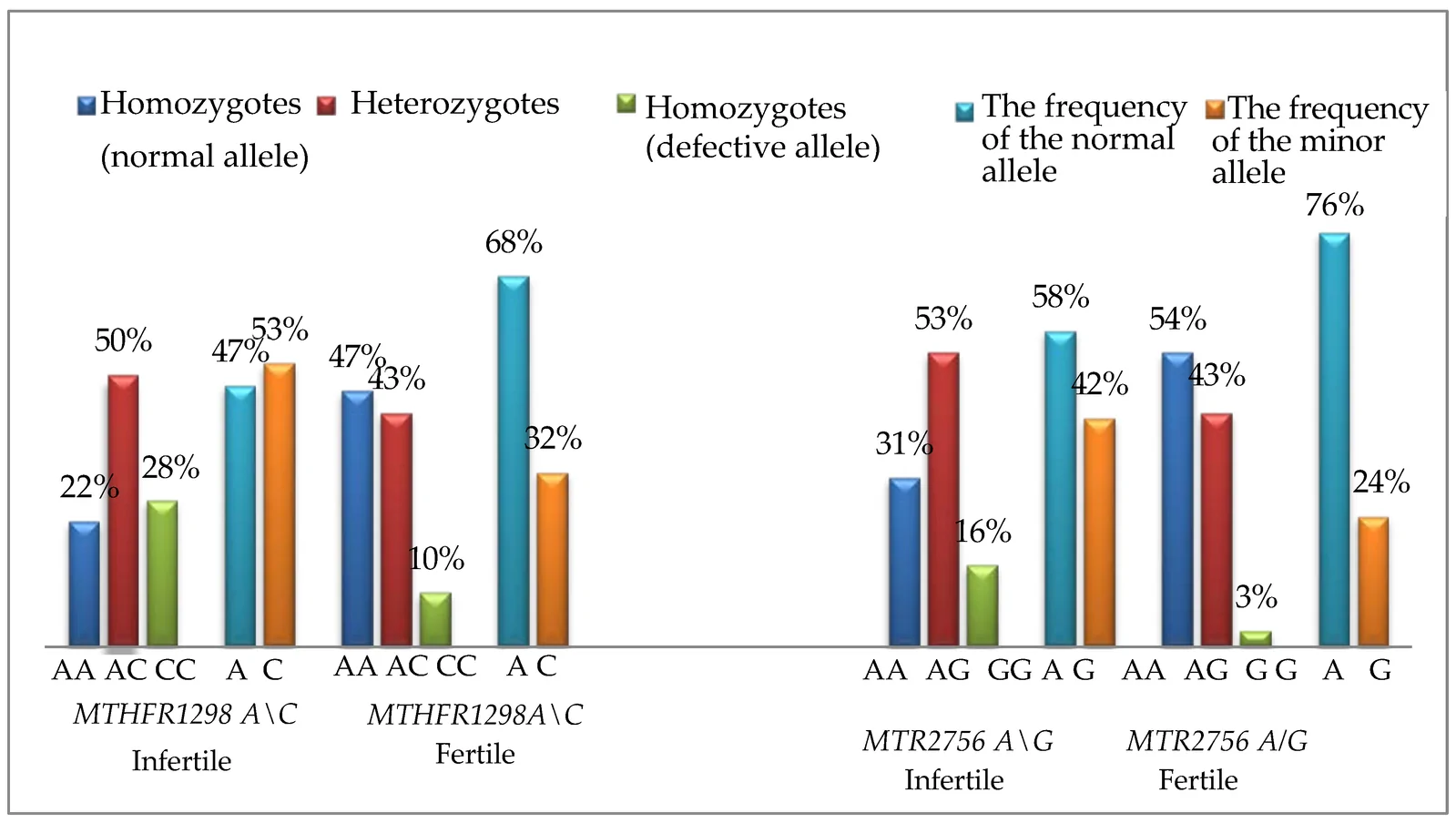
The distribution frequencies of the genotypes and alleles of the A1298C *MTHFR* and A2756G *MTR* loci in the subgroup of men with asthenozoospermia and fertile men.

**Figure 4:**
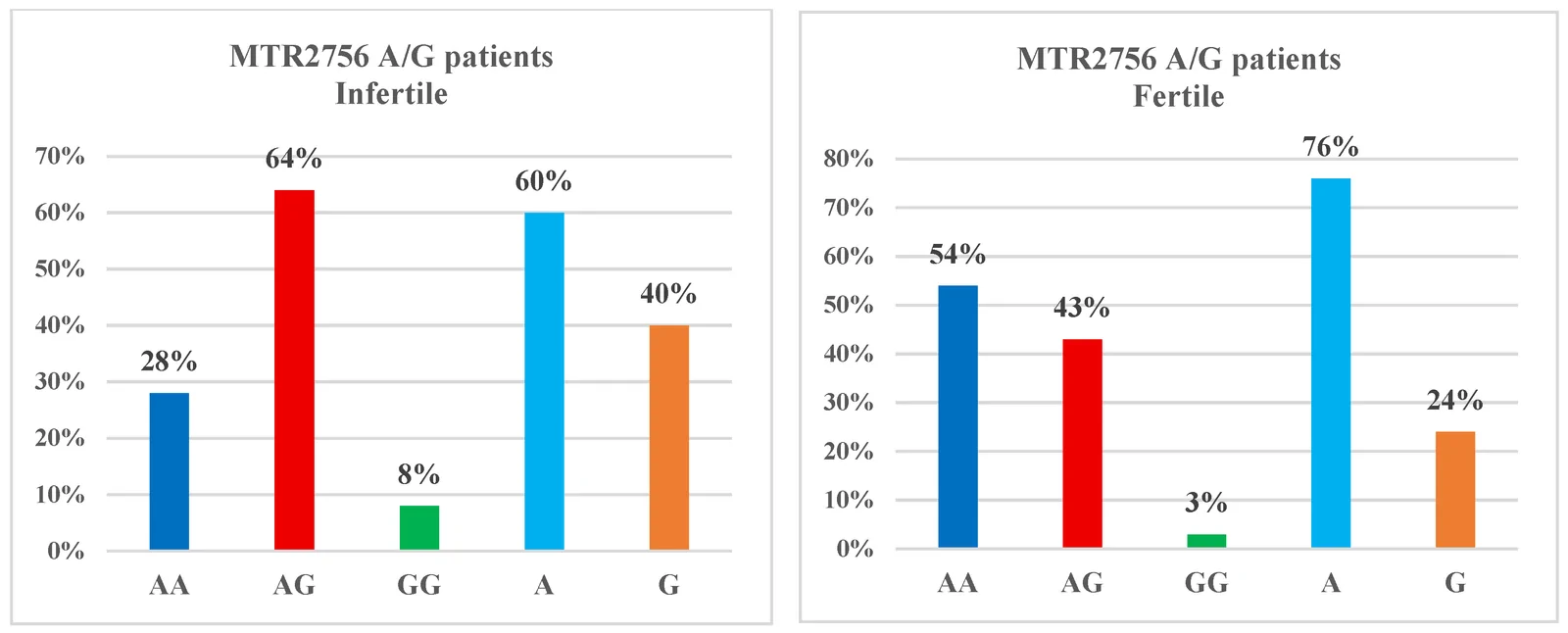

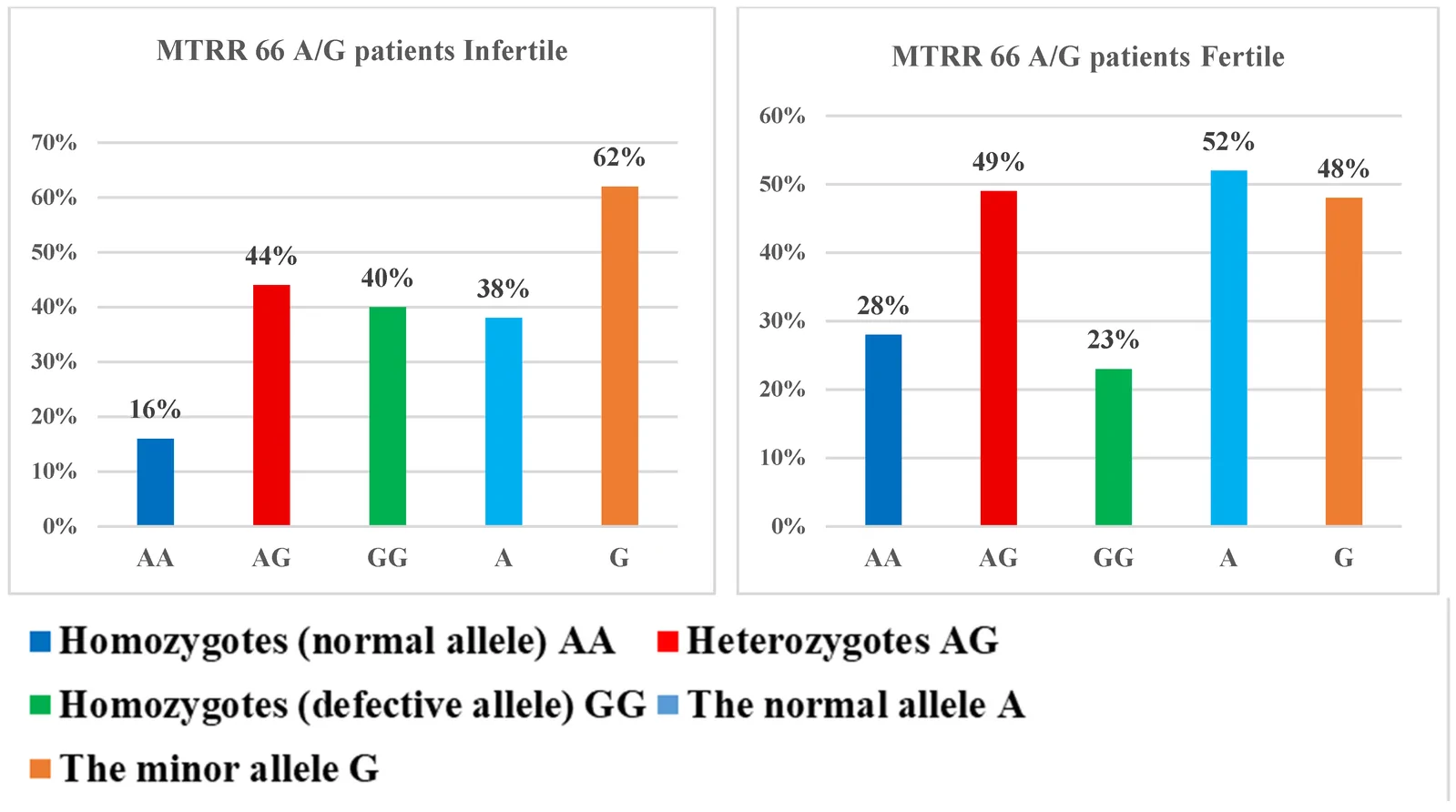
The frequency of distribution of genotypes and alleles by polymorphisms A2756G *MTR* and A66G *MTRR* in the subgroup of men with teratozoospermia and fertile men.

**Figure 5:**
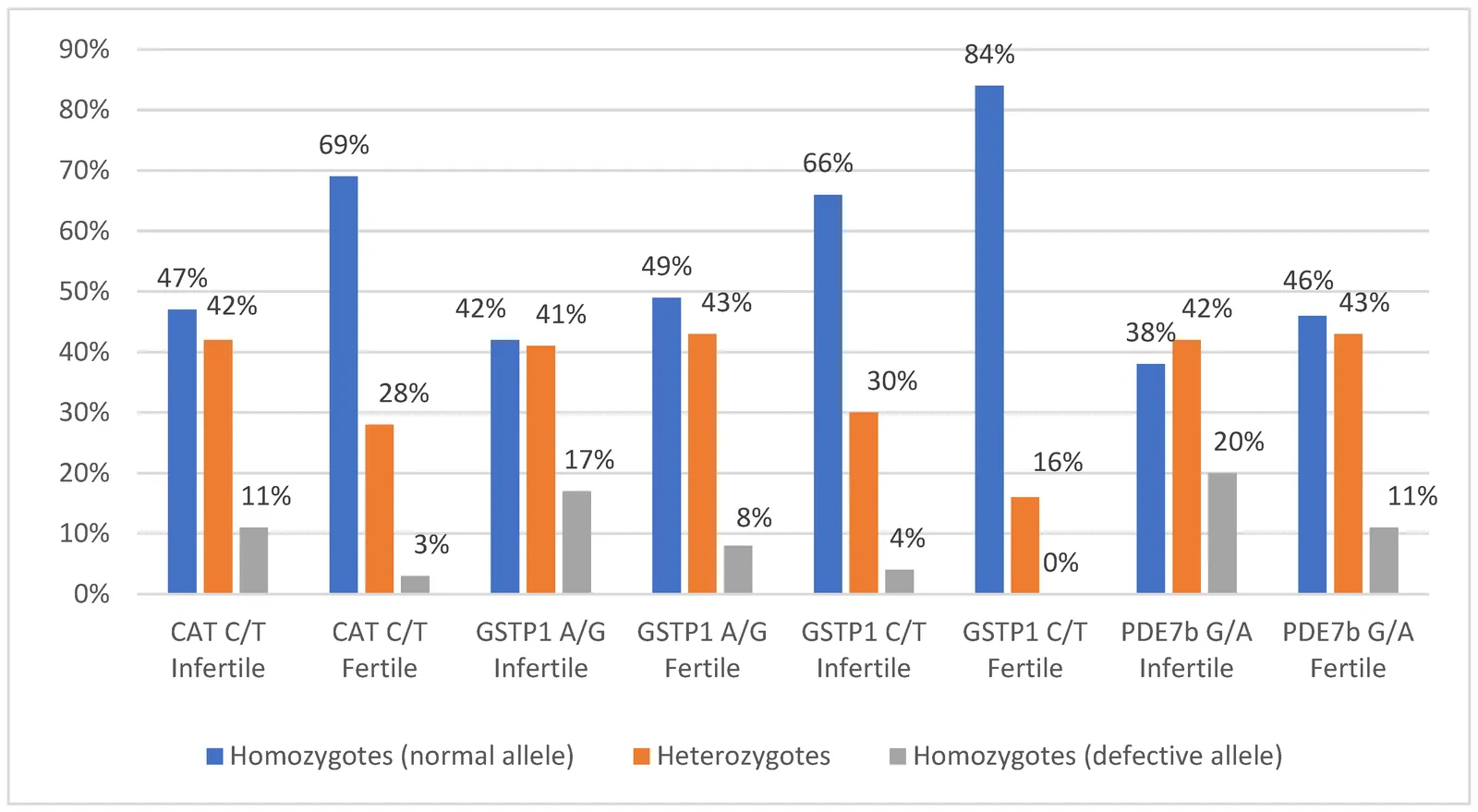
Distribution of genotype frequencies by *CAT*-262C > T, *GSTP1* Ile105Val (A > G), *GSTP1* Ala114Val (C > T), and *PDE7B* G > A polymorphism in patients with impaired semen parameters and fertile men.

**Figure 6:**
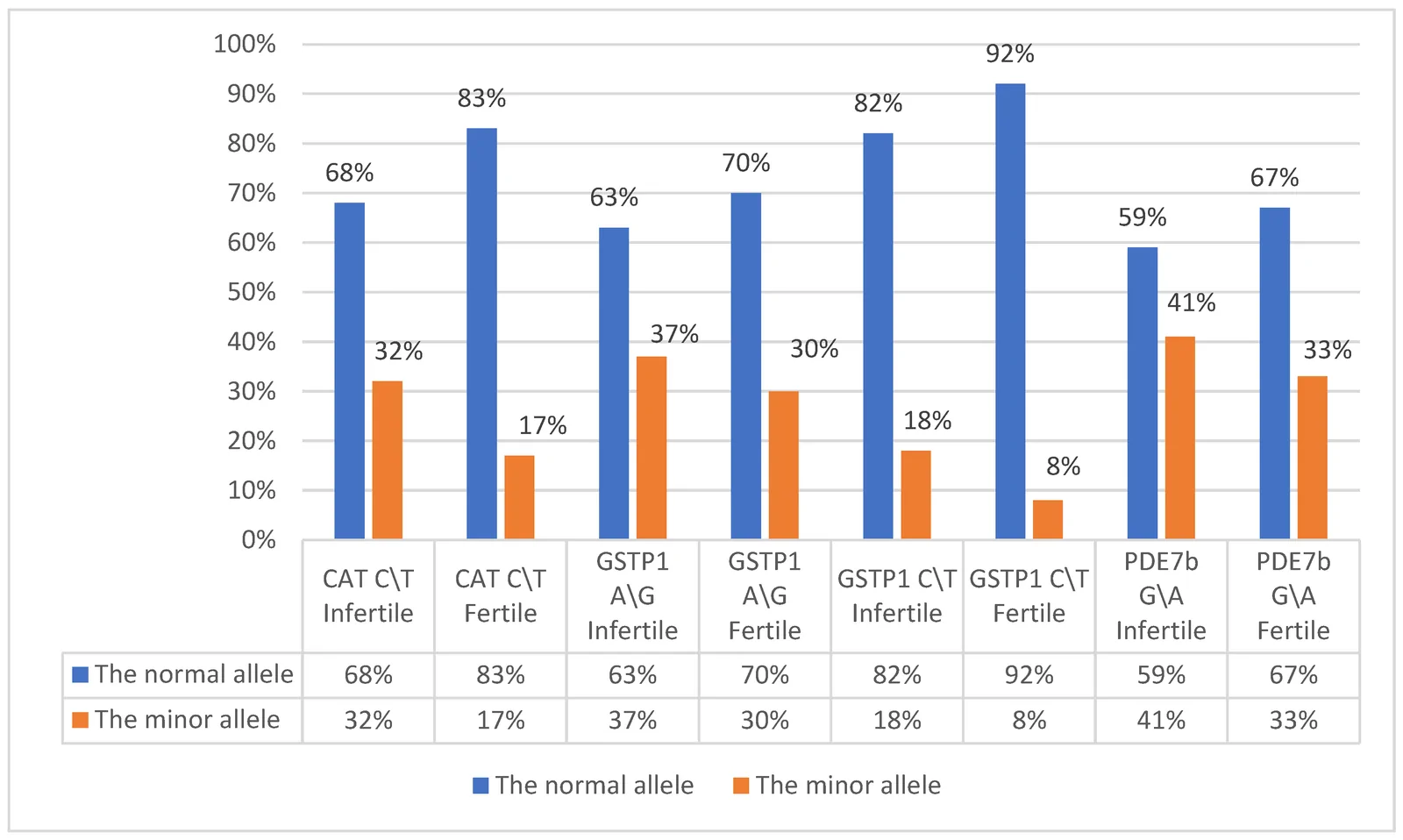
Distribution of allelic variants of the antioxidant protection genes *CAT* and *GSTP1* and phosphodiesterase *PDE7B* in groups of patients with impaired semen parameters and fertile men.

**Figure 7:**
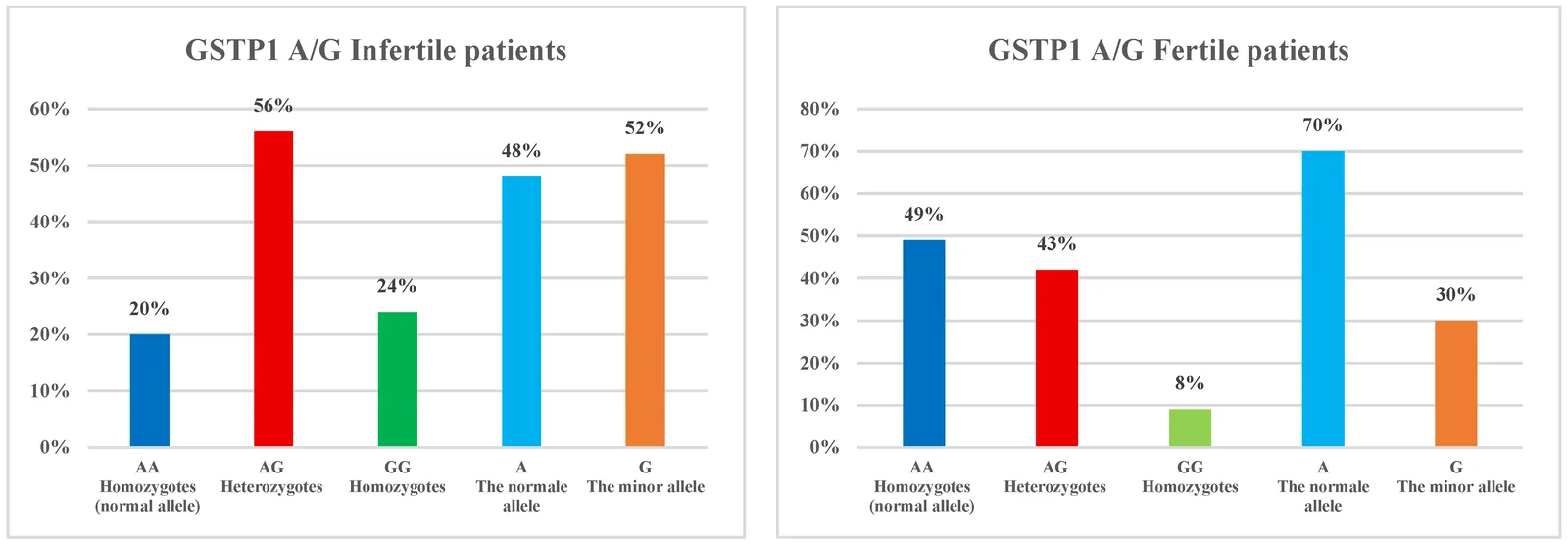
Frequency distribution of allele variants and genotypes of the 105Ile/Val (AG) polymorphism of the *GSTP1* (rs1695) gene among infertile men with teratozoospermia and in the group of fertile men.

**Figure 8:**
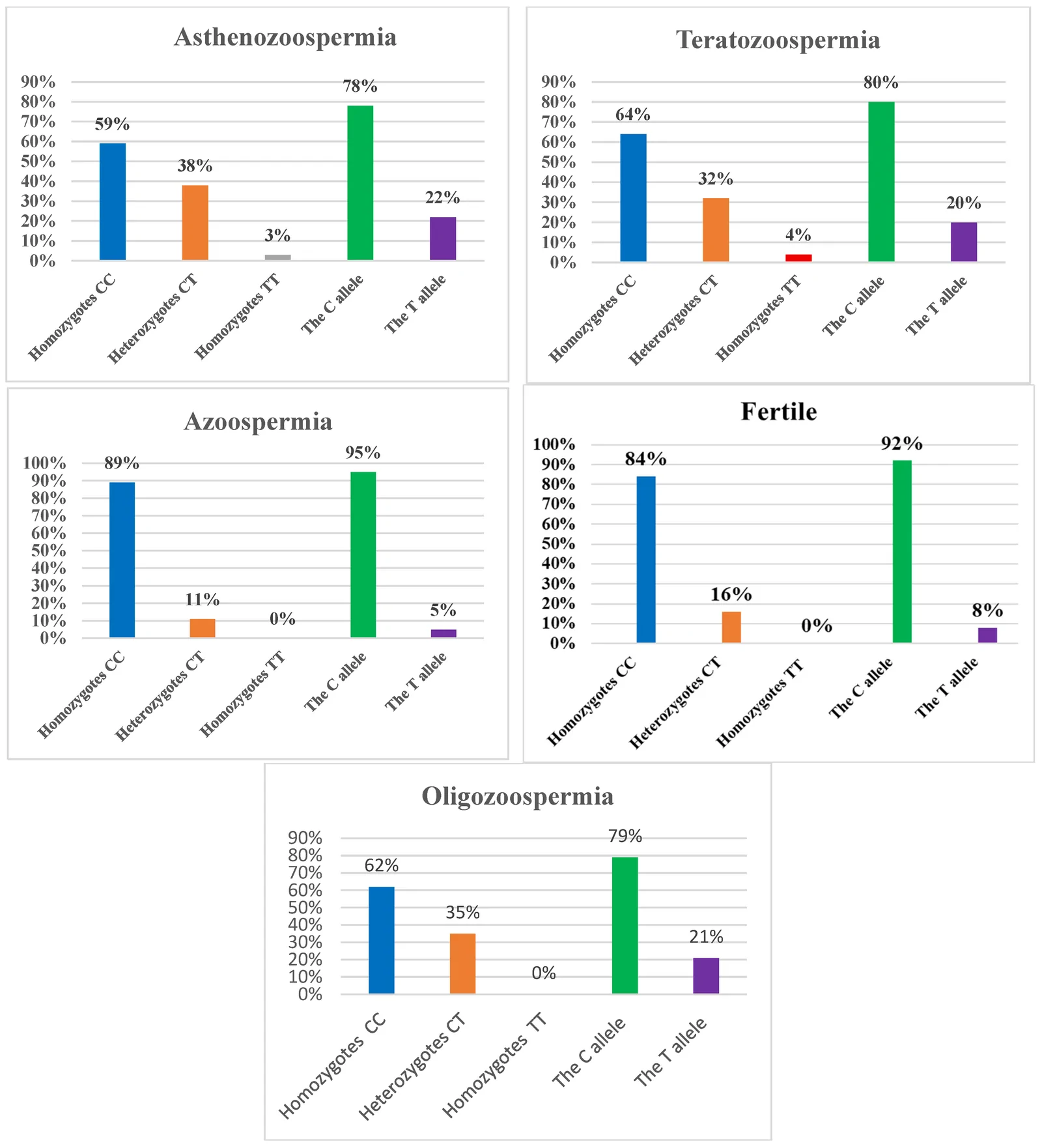
Distribution of gene frequencies and genotypes by polymorphism Ala114Val (C\T) *GSTP1* (rs1138272) in subgroups of men with different forms of impaired semen parameters and fertile men.

**Figure 9:**
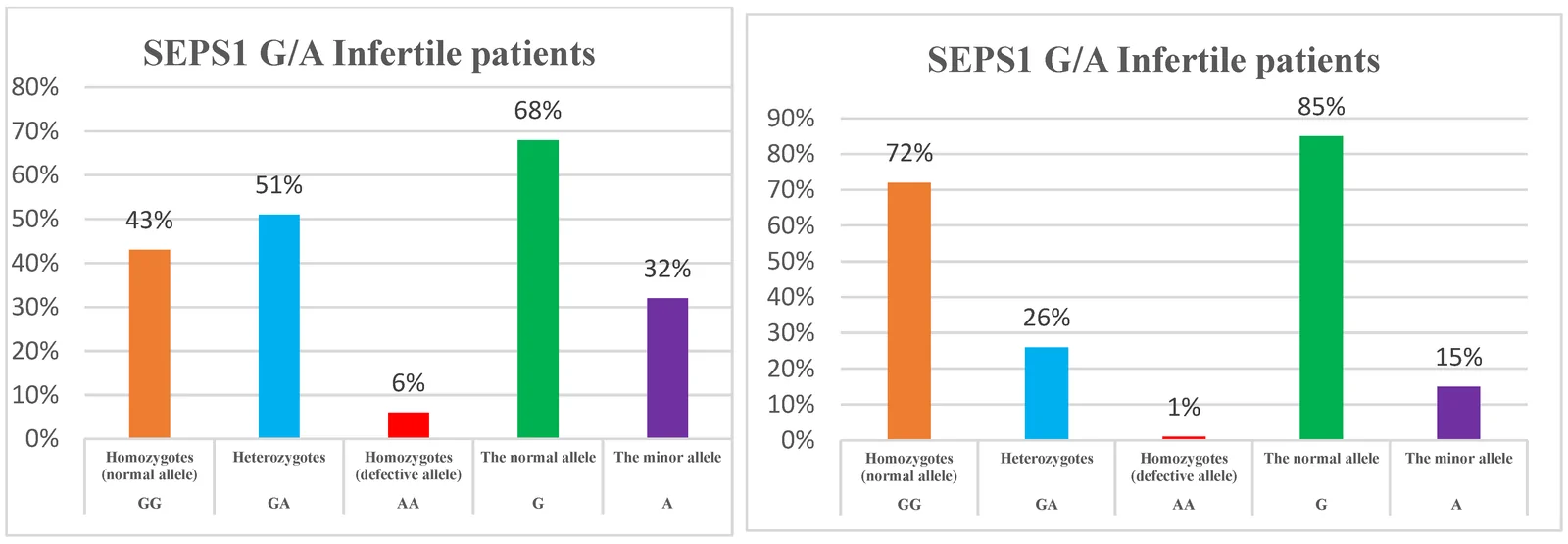
Frequencies of alleles and genotypes of the G-105A polymorphism of the *SEPS1* gene in infertile men with impaired semen parameters and fertile men.

**Figure 10:**
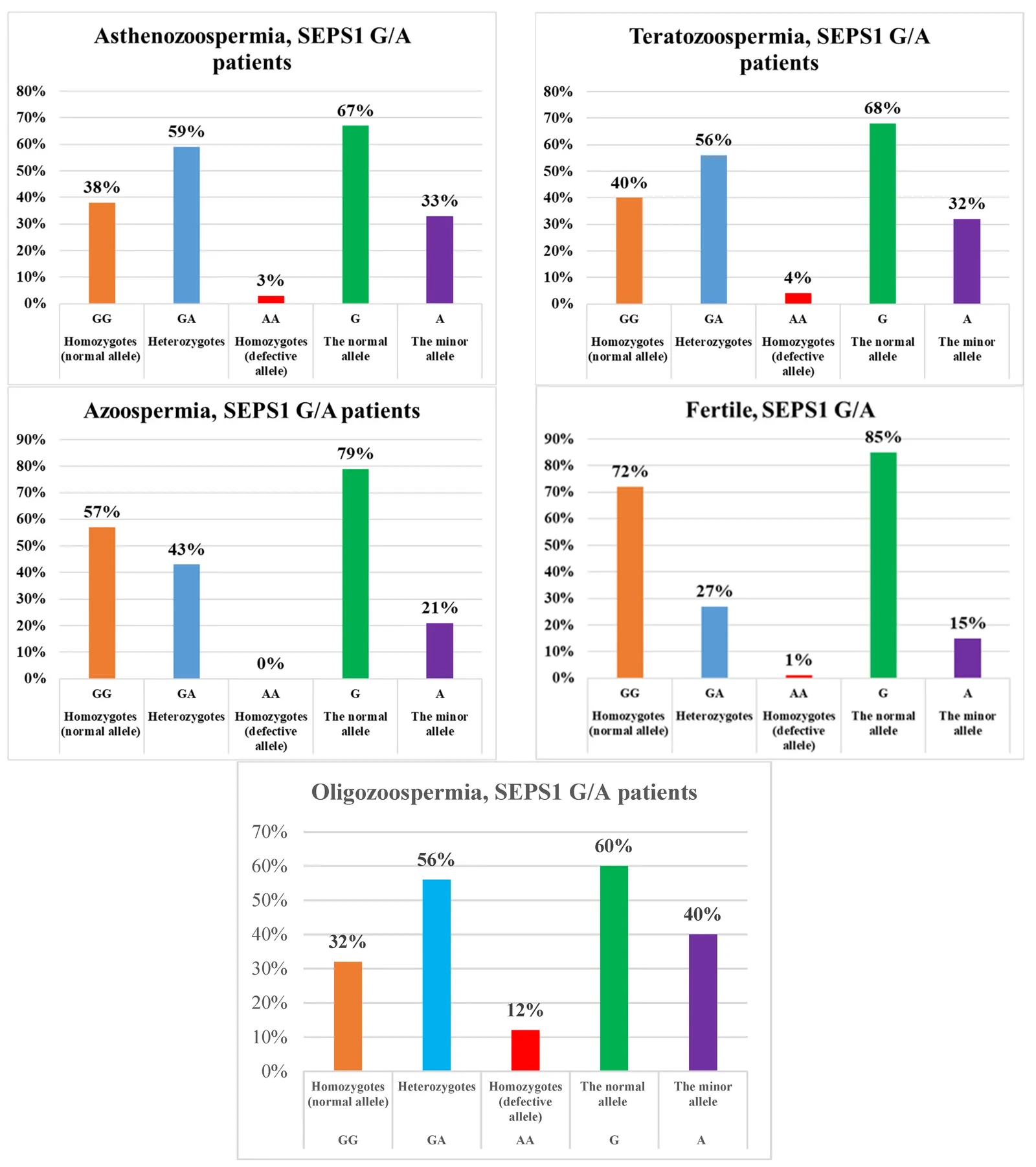
The frequency of distribution of genotypes and allelic variants of G/A *SEPS1* in the group of fertile men and in subgroups of men with various forms of pathospermia.

**Figure 11:**
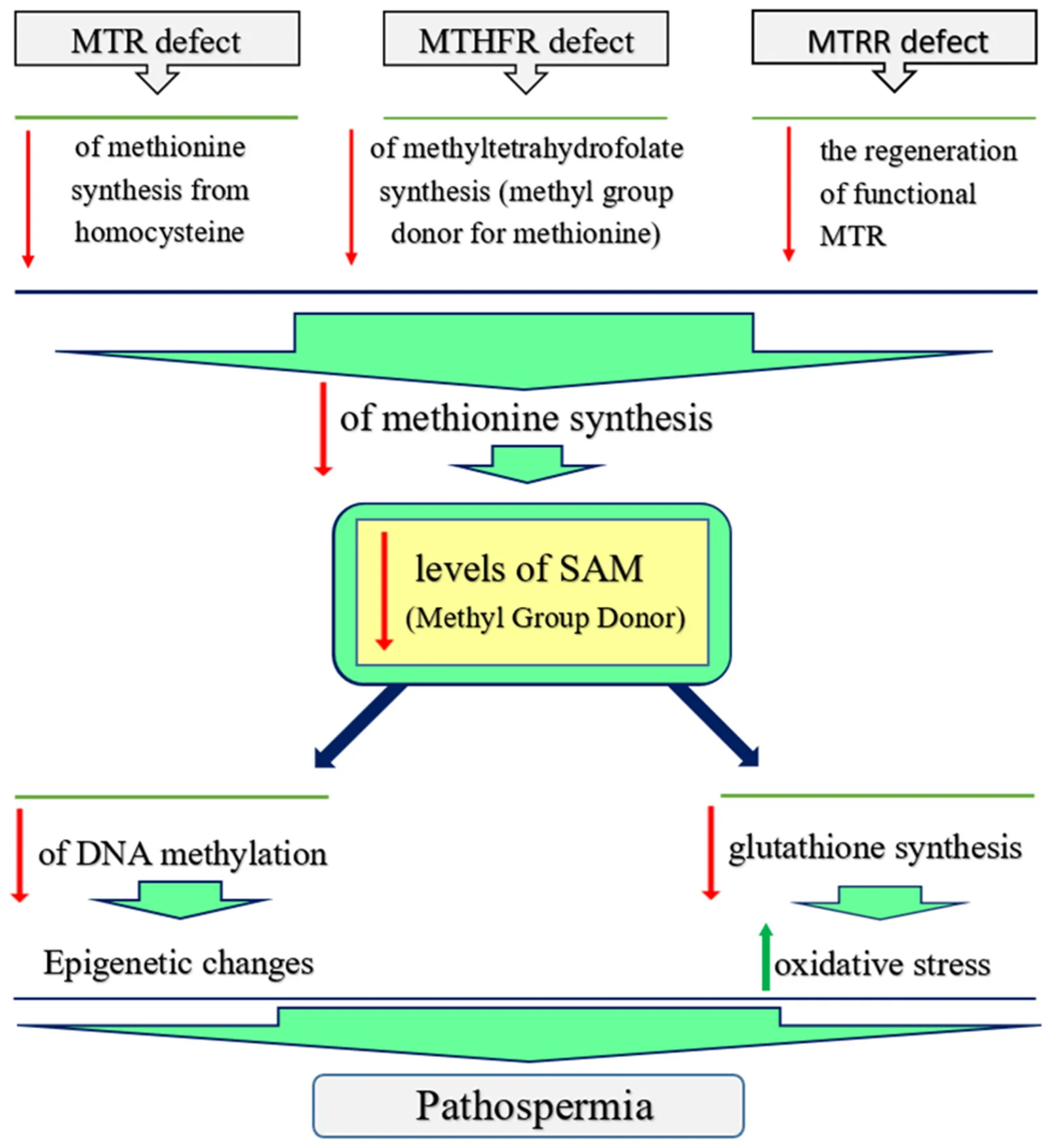
Features of the action of defective folate cycle genes and their relationship to pathospermia. Red downward arrows indicate a decrease, and the green upward arrow indicates an increase in the corresponding process or parameter. Green and dark blue arrows show the direction of the cascade from the initial gene defects to the resulting impairment of semen parameters.

**Table 1:** Baseline clinical and demographic characteristics of the study groups.

Parameter	Fertile (*n* = 68)	Infertile Overall (*n* = 119)	*p*-Value *	Asthenozoospermia (*n* = 32)	Teratozoospermia (*n* = 25)	Oligozoospermia (*n* = 34)	Azoospermia (*n* = 28)	*p*-Value **
Age, years (M ± SD)	31.4 ± 5.2	32.1 ± 5.8	0.431	31.8 ± 5.5	32.5 ± 6.1	31.6 ± 5.4	32.0 ± 5.9	0.892
Duration of infertility, years (M ± SD)	-	4.2 ± 2.1	-	4.0 ± 1.9	4.5 ± 2.3	4.2 ± 2.1	4.1 ± 2.0	0.567
BMI, kg/m^2^ (M ± SD)	25.3 ± 3.1	25.8 ± 3.4	0.342	25.6 ± 3.2	26.1 ± 3.6	25.4 ± 3.0	25.7 ± 3.3	0.781
**Hormonal parameters (M ± SD):**								
FSH, IU/L	5.8 ± 2.1	6.4 ± 4.2	0.287	5.9 ± 2.8	6.1 ± 3.1	6.2 ± 3.0	7.2 ± 5.9	0.412
LH, IU/L	4.2 ± 1.8	4.6 ± 2.9	0.324	4.3 ± 2.1	4.5 ± 2.4	4.4 ± 2.2	5.0 ± 3.8	0.536
Testosterone, nmol/L	16.8 ± 5.3	15.9 ± 6.1	0.345	16.2 ± 5.8	15.7 ± 6.3	16.1 ± 5.7	15.8 ± 6.4	0.923
Prolactin, mIU/L	284 ± 112	302 ± 138	0.396	295 ± 125	308 ± 142	292 ± 128	305 ± 148	0.912

* *p*-value for comparison between fertile and infertile men (overall group) using Student’s *t*-test for continuous variables. ** *p*-value for comparison among the three subgroups of infertile men using one-way analysis of variance (ANOVA) for continuous variables. All *p* > 0.05—groups comparable. M ± SD—arithmetic mean ± standard deviation. FSH—follicle-stimulating hormone; LH—luteinizing hormone. Normal reference values: FSH 1.5–12.4 IU/L; LH 1.7–8.6 IU/L; testosterone 8.64–29.0 nmol/L; prolactin 86–324 mIU/L.

**Table 2:** Post-hoc statistical power for detecting genetic associations in each infertile male subgroup.

Subgroup	*n*	Observed OR (For the Strongest Associated SNP)	Achieved Power (α = 0.05, Two-Sided)	Minimum OR Detectable with 80% Power *
All infertile	119	2.34 (for *CAT*-262T)	63.8%	2.73
Asthenozoospermia	32	2.54 (for *CAT*-262T CT + TT)	57.1%	3.38
Teratozoospermia	25	2.91 (for *GSTP1* Ala114Val)	53.6%	4.22
Oligozoospermia	34	3.12 (for *CAT*-262T T-allele)	67.5%	3.70
Azoospermia	28	1.93 (for *SEPS1* GA + AA)	29.9%	3.60

* Power calculations assumed an additive genetic model and used the observed risk allele frequency in the control group for each SNP. Conventional acceptable power is ≥80%; values below this indicate that only large effects could be reliably detected, whereas moderate or small effects may have been missed. These estimates highlight the exploratory nature of the subgroup analyses.

**Table 3:** Allele and genotype distribution of folate cycle genes in infertile vs. fertile men.

SNP	Genotypes and Alleles	Infertile, % (*n* = 119)	Fertile, % (*n* = 68)	χ^2^	Odds Ratio	95% CI	*p* Value	q (FDR)
***MTHFR677 C\T***	CC	0.42 (50)	0.49 (33)	0.41	1.18	0.61–2.28	0.650	0.75
	CT	0.41 (49)	0.38 (26)					
	TT	0.17 (20)	0.13 (9)					
	CT + TT	0.58 (69)	0.51 (35)	0.831	1.30	0.72–2.53	0.389	0.53
	C-allele	0.63	0.68	0.96	1.25	0.82–2.12	0.327	0.46
	T-allele	0.37	0.32					
***MTHFR1298 A\C***	AA	0.36 433)	0.47 (32)	3.345	1.89	0.75–4.76	0.184	0.85
	AC	0.44 (52)	0.43 (29)					
	CC	0.20 (24)	0.10 (7)					
	AC + CC	0.64 (76)	0.53 (36)	2.112	1.58	0.85–2.93	0.146	0.85
	A-allele	0.58	0.68	3.128	1.56	0.96–2.54	0.077	0.77
	C-allele	0.42	0.32					
***MTR2756 A\G***	AA	0.36 (43)	0.54 (37)	8.912	4.12	1.38–12.31	0.012 *	0.12
	AG	0.48 (57)	0.43 (29)					
	GG	0.16 (19)	0.03 (2)					
	AG + GG	0.64 (76)	0.46 (31)	5.401	2.10	1.12–3.94	0.020 *	0.20
	A-allele	0.60	0.76	7.892	2.12	1.27–3.54	0.005 *	0.05 (q = 0.12 after FDR)
	G-allele	0.40	0.24					
***MTRR66A\G***	AA	0.20 (24)	0.28 (19)	3.109	1.89	0.78–4.58	0.210	0.85
	AG	0.43 (50)	0.49 (33)					
	GG	0.38 (45)	0.23 (16)					
	AG + GG	0.80 (95)	0.72 (49)	1.502	1.57	0.75–3.29	0.220	0.85
	G-allele	0.59	0.48	3.742	1.58	0.99–2.51	0.053	0.53
	A-allele	0.41	0.52					

* Statistically significant differences between the groups (*p* < 0.05); All SNPs were in Hardy–Weinberg equilibrium in both fertile and infertile groups (*p* > 0.05 for each comparison).

**Table 4:** Distribution of genotype and allele frequencies by folate cycle gene polymorphisms in patients with various forms of sperm parameter disorders.

SNP	Genotypes	Fertile, % (*n* = 68)	Asthenozoospermia % (*n* = 32)	χ^2^/*p*	Teratozoospermia, % (*n* = 25)	χ^2^/*p*	Oligozoospermia, % (*n* = 34)	χ^2^/*p*	Azoospermia, % (*n* = 28)	χ^2^/*p*
***MTHFR 677 C\T***	CC	0.49 (33)	0.47 (15)	0.10/0.95	0.40 (10)	1.54/ 0.464	0.32 (11)	5.21/0.074	0.50 (14)	1.38/ 0.50
	CT	0.38 (26)	0.38 (12)		0.52 (13)		0.47 (16)		0.29 (8)	
	TT	0.13 (9)	0.16 (5)		0.08 (2)		0.21 97)		0.21 (6)	
	CT + TT	0.51 (35)	0.53 (17)	0.02/ 0.88	0.60 (15)	0.53/ 0.47	0.68 (23)	4.12/0.042	0.50 (14)	0.02 /0.9
	C	0.68	0.66	0.08/ 0.78	0.66	0.04/ 0.83	0.56	4.89/0.027	0.64	0.20/ 0.65
	T	0.32	0.34		0.34		0.54		0.36	
***MTHFR 1298 A\C***	AA	0.47 (32)	0.22 (7)	8.12/ 0.017 *	0.48 (12)	1.86/ 0.4	0.29 (10)	4.67/0.97	0.46 (13)	0.33/ 0.85
	AC	0.43 (29)	0.50 (16)		0.32 (8)		0.50 (17)		0.39 (11)	
	CC	0,10 (7)	0.28 (9)		0.20 (5)		0.21 (7)		0.14 (4)	
	AC + CC	0.53 (36)	0.78 (25)	5.80/ 0.016 *	0.52 (13)	0.01 /0.936	0.71 (24)	3.98/0.046	0.54 (15)	0.0032 /0.95
	A	0.68	0.47	8.50/ 0.004 *	0.64	0.32/ 0.572	0.54	4.12/0.042	0.66	0.097 /0.76
	C	0.32	0.53		0.36		0.46		0.34	
***MTR 2756 A\G***	AA	0.54 (37)	0.31 (10)	8.00/ 0.018 *	0.28 (7)	5.51/ 0.064	0.26 (9)	8.45/0.015	0.61 (17)	5.87/ 0.053
	AG	0.43 (29)	0.53 (17)		0.64 (16)		0.56 (19)		0.25 (7)	
	GG	0.03 (2)	0.16 (5)		0.08 (2)		0.18 (96)		0.14 (4)	
	AG + GG	0.46 (31)	0.69 (22)	4.69/ 0.030 *	0.72 (18)	5.12/ 0.024 *	0.74 (25)	7.12/0.008	0.39 (11)	0.32/ 0.57
	A	0.76	0.58	6.66/0.010 *	0.60	4.44/ 0.035 *	0.54	9.02/0.003	0.73	0.134/ 0.714
	G	0.24	0.42		0.40		0.46		0.27	
***MTRR 66 A\G***	AA	0.28 (19)	0.22 (7)	4.26/ 0.119	0.16 (4)	2.91/ 0.23	0.18 (6)	1.98/0.37	0.21 (6)	0.533/ 0.766
	AG	0.49 (33)	0.34 (11)		0.44 (11)		0.44 (15)		0.50 (14)	
	GG	0.23 (16)	0.44 (14)		0.40 (10)		0/38 (13)		0.29 (8)	
	AG + GG	0.72 (49)	0.78 (25)	0.42/ 0.52	0.84 (21)	1.40/ 0.24	0.82 (28)	1.21/0.27	0.79 (22)	0.437/ 0.509
	A	0.52	0.39	3.01/ 0.083	0.38	2.95/ 0.086	0.40	2/89/0.089	0.46	0.53/ 0.467
	G	0.48	0.61		0.62		0.60		0.54	

* Statistically significant differences between the groups (*p* < 0.05); All SNPs were in Hardy–Weinberg equilibrium in both fertile and infertile groups (*p* > 0.05 for each comparison).

**Table 5:** Distribution of genotypes and alleles by polymorphisms *CAT* C262T, *GSTP1* 105Ile/Val (AG), 114Ala/Val (CT) and *PDE7B* G/A among fertile and infertile men with impaired semen parameters.

SNP	Genotypes and Alleles	Infertile, % (*n* = 119)	Fertile, % (*n* = 68)	χ^2^	Odds Ratio	95% CI	*p*	q (FDR)
***CAT-262C > T***	CC	0.47 (56)	0.69 (47)	6.901	4.12	0.88–18.40	0.008 *	0.08
	CT	0.42 (50)	0.28 (19)					
	TT	0.11 (13)	0.03 (2)					
	CT + TT	0.53 (63)	0.31 (21)	8.112	2.51	1.32–4.78	0.004 *	0.04
	C-allele	0.68	0.83	8.023	2.31	1.32–4.04	0.005 *	0.05 (q = 0.05)
	T-allele	0.32	0.17					
***GSTP1 Ile105Val***	(Ile/Ile) AA	0.42 (50)	0.49 (33)	2.333	1.89	0.66–5.41	0.312	0.85
	(Ile/Val) AG	0.41 (49)	0.43 (29)					
	(Val/Val)GG	0.17 (20)	0.08 (6)					
	(Ile + Val) AG + GG	0.58 (69)	0.51 (35)	0.843	1.34	0.72–2.52	0.359	0.85
	A-allela	0.63	0.70	1.789	1.36	0.85–2.18	0.181	0.85
	G-allele	0.37	0.30					
***GSTP1 Ala114Val***	(Ala/Ala) CC	0.66(79)	0.84 (57)	5.341	3.12	0.95–10.2	0.022	0.22
	(Ala/Val) CT	0.30 (36)	0.16 (11)					
	(Val/Val) TT	0.04 (4)	0 (0)					
	(Ala + Val) CT + TT	0.34 (40)	0.16 (11)	6.723	2.68	1.25–5.74	0.010	0.10
	C-allele	0.82	0.92	6.802	2.55	1.26–5.18	0.009	0.09
	T-allele	0.18	0.08					
***PDE7BG\A***	GG	0.38 (45)	0.46 (31)	1.334	1.65	0.63–4.33	0.512	0.85
	GA	0.42 (50)	0.43 (29)					
	AA	0.20 (24)	0.11 (8)					
	GA + AA	0.62 (74)	0.54 (37)	1.102	1.38	0.73–2.61	0.294	0.85
	G-allele	0.59	0.67	2.101	1.43	0.89–2.29	0.147	0.85
	A-allele	0.41	0.33					

* Statistically significant differences between the groups (*p* < 0.05); All SNPs were in Hardy–Weinberg equilibrium in both fertile and infertile groups (*p* > 0.05 for each comparison).

**Table 6:** The frequency of occurrence of genotypes and allelic variants of the *CAT*, *GSTP1*, and *PDE7B* genes in subgroups of infertile men with different forms of impaired semen parameters.

SNP	Genotype/ Subgroups	Fertile, % (*n* = 68)	Asthenozoospermia,% (*n* = 32)	χ^2^/*p*	Teratozoospermia, % (*n* = 25)	χ^2^/*p*	Oligozoospermia, % (*n* = 34)	χ^2^/*p*	Azoospermia,% (*n* = 28)	χ^2^/*p*
***CAT C\T***	CC	0.69 (47)	0.47 (15)	7.38/0.025 *	0.40 (10)	6.69/0.035 *	0.35 (12)	10.12/0.006	0.64 (18)	0.31/0.58
	CT	0.28 (19)	0.38 (12)		0.52 (13)		0.53 (18)		0.32 (9)	
	TT	0.02 (2)	0.16 (5)		0.08 (2)		0/12 (4)		0.04 (1)	
	CT + TT	0.30 (21)	0.53 (17)	4.57/0.033 *	0.60 (15)	6.53/0.011 *	0.65 (22)	11.23/0.0008	0.36 (10)	0.21/0.65
	C	0.83	0.66	7.61/0.006 *	0.66	6.32/0.012 *	0.62	12.01/0.0005	0.80	0.18/0.67
	T	0.17	0.34		0.34		0.38		0.20	
***GSTP1 Ile105Val***	(Ile/Ile) AA	0.49 (33)	0.53 (17)	1.73/0.42	0.20 (5)	7.61/0.022 *	0.32 (11)	3.12/0.21	0.50 (14)	0.01/0.92
	(Ile/Val)AG	0.43 (29)	0.31 (10)		0.56 (14)		0.50 (17)		0.36 (10)	
	(Val/Val)GG	0.08 (6)	0.16 (5)		0.24 (6)		0.18 (6)		0.14 (4)	
	(Ile + Val) AG + GG	0.51 (35)	0.47 (15)	0.18/0.67	0.80 (20)	6.16/0.013 *	0.68 (23)	2.98/0.084	0.50 (14)	0.01/0.92
	A	0.70	0.69	0.025/0.87	0.48	7.58/0.006 *	0.57	4.12/0.042	0.68	0.06/0.81
	G	0.30	0.31		0.52		0.43		0.32	
***GSTP Ala114Val***	(Ala/Ala) CC	0.84 (57)	0.59 (19)	8.14/0.017 *	0.64 (16)	5.87/0.053	0.62 (21)	6.78/0.034	0.89 (25)	0.45/0.50
	(Ala/Val) CT	0.16 (11)	0.38 (12)		0.32 (8)		0.35 (12)		0.11 (3)	
	(Val/Val) TT	0 (0)	0.03 (1)		0.04 (1)		0.03 (1)		0.0 (0)	
	(Ala + Val) CT + TT	0.16 (11)	0.41 (13)	7.13/0.008 *	0.36 (9)	4.26/0.039 *	0.38 (13)	6.12/0.013	0.11 (3)	0.39/0.53
	C	0.92	0.78	7.56/0.006 *	0.80	5.18/0.023 *	0.79	6.45/0.011	0.95	0.36/0.55
	T	0.08	0.22		0.20		0.21		0.05	
***PDE7B***	GG	0.46 (31)	0.34 (11)	1.16/0.56	0.52 (13)	2.06/0.36	0.32 (11)	1.89/0.39	0.29 (8)	2.21/0.14
	GA	0.43 (29)	0.50 (16)		0.28 (7)		0.50 (17)		0.50 (14)	
	AA	0.11 (8)	0.16 (5)		0.20 (5)		0.18 (6)		0.21 (6)	
	GA + AA	0.54 (37)	0.66 (21)	1.12/0.29	0.48 (12)	0.30/0.58	0.68 (23)	1.89/0.17	0.71 (20)	2.28/0.13
	Aллeль G	0.67	0.59	1.08/0.30	0.66	0.014/0.907	0.57	1.98/0.16	0.54	1.46/0.23
	AллeльA	0.33	0.41		0.34		0.43		0.46	

* Statistically significant differences between the groups (*p* < 0.05); All SNPs were in Hardy–Weinberg equilibrium in both fertile and infertile groups (*p* > 0.05 for each comparison).

**Table 7:** Frequencies of genotype distribution by G-105A *SEPS1* polymorphism in infertile and fertile men.

*SEPS1*			χ^2^	Odds Ratio	95% CI	*p*	q (FDR)
The *SEPS1* G\A Genotype	Infertile Men % (*n* = 119)	Fertile Men % (*n* = 68)					
GG	0.43 (51)	0.72 (49)	14.23	2.98	1.45–6.12	0.0002 *	0.002
GA	0.51 (61)	0.27 (18)					
AA	0.06 (7)	0.01 (1)					
Carriers (GA + AA genotypes)	0.57 (68)	0.28 (19)	14.89	3.45	1.82–6.54	0.0001 *	0.001
Frequency of the G allele	0.68	0.85	12.34	2.67	1.58–4.51	0.0004 *	0.004
Frequency of the A allele	0.32	0.15					

* Statistically significant differences between the groups (*p* < 0.05).

**Table 8:** Distribution frequencies of polymorphic variants of the G-105A *SEPS1* locus (rs28665122) in subgroups of infertile patients and among fertile men.

Genotype/Allele	Fertile, % (*n* = 68)	Asthenozoospermia, % (*n* = 32)	χ^2^/*p*	Teratozoospermia, % (*n* = 25)	χ^2^/*p*	Oligozoospermia, % (*n* = 34)	χ^2^/*p*	Azoospermia, % (*n* = 28)	χ^2^/*p*
GG	0.72 (49)	0.38 (12)	10.93/0.0042 *	0.40 (10)	8.14/0.017 *	0.32 (11)	16.37/0.0002 *	0.57 (16)	2.77/0.25
GA	0.27 (18)	0.59 (19)		0.56 (14)		0.56 (19)		0.43 (12)	
AA	0.01 (1)	0.03 (1)		0.04 (1)		0.12 (4)		0.00 (0)	
GA + AA	0.28 (19)	0.62 (20)	10.92/0.00095 *	0.60 (15)	8.10/0.0044 *	0.68 (23)	14.89/0.0003 *	0.43 (12)	2.01/0.15
G-allele	0.85	0.67	8.88/0.0028 *	0.68	7.005/0.0081 *	0.60	12.34/0.0001 *	0.79	1.29/0.256
A-allele	0.15	0.33		0.32		0.40		0.21	

* Statistically significant differences between the groups (*p* < 0.05).

**Table 9:** Multivariate logistic regression analysis of risk factors for impaired semen parameters.

Factor	Unadjusted OR (95% CI)	*p*	Adjusted OR * (95% CI)	*p*	q (FDR)
*MTR* A2756G (G allele)	1.82 (1.09–3.04)	0.022 *	1.78 (1.05–3.02)	0.031 *	0.31 *
*MTHFR* A1298C (C allele)	1.56 (0.96–2.54)	0.077	1.52 (0.92–2.51)	0.102	0.51
*CAT*-262T (T allele)	2.31 (1.32–4.04)	0.005 *	2.34 (1.34–4.08)	0.002 *	0.04 *
*GSTP1* Ala114Val (T allele)	2.55 (1.26–5.18)	0.009 *	2.41 (1.18–4.92)	0.016 *	0.16
*SEPS1* G-105A (A allele)	2.67 (1.58–4.51)	0.0004 *	2.89 (1.78–4.69)	0.0001	0.002 *
Age (per 1 year)	1.02 (0.96–1.09)	0.431	1.01 (0.95–1.09)	0.748	0.85
BMI (per 1 kg/m^2^)	1.05 (0.94–1.18)	0.342	1.04 (0.93–1.17)	0.493	0.85

* Adjusted for age, BMI, levels of FSH, LH, testosterone, and prolactin.

**Table 10:** Analysis of interactions between polymorphisms (epistasis).

Interaction	β-Coefficient	Standard Error	*p*	OR (95% CI)	q (FDR)
*MTR* A2756G × *CAT*-262T	0.54	0.32	0.091	1.72 (0.92–3.22)	0.85
*MTR* A2756G × *SEPS1* G-105A	0.48	0.29	0.098	1.62 (0.92–2.86)	0.85
*CAT*-262T × *GSTP1* Ala114Val	0.61	0.34	0.073	1.84 (0.94–3.60)	0.85
*SEPS1* G-105A × *GSTP1* Ala114Val	0.39	0.31	0.208	1.48 (0.81–2.70)	0.85

**Table 11:** Comparison of genetic risk score (GRS) between groups.

Group	*n*	GRS (M ± SD)	Median (Q1–Q3)	*p* *	q (FDR)
Fertile	68	1.8 ± 1.1	2 (1–2)	-	-
Infertile (overall group)	119	3.1 ± 1.4	3 (2–4)	<0.001	<0.001
Asthenozoospermia	32	3.1 ± 1.2	3 (2–4)	<0.001	<0.001
Teratozoospermia	25	3.2 ± 1.4	3 (2–4)	<0.001	<0.001
Oligozoospermia	34	3.4 ± 1.5	4 (3–5)	<0.001	<0.001
Azoospermia	28	2.3 ± 1.1	2 (2–3)	0.087	0.087

* Comparison with the group of fertile men (Mann–Whitney U test).

**Table 12:** Results of ROC analysis for individual polymorphisms and combination of polymorphisms.

Predictor	AUC	95% CI	Sensitivity, %	Specificity, %	Cut-Off Value
*MTR* A2756G (G allele)	0.58	0.49–0.67	47.1	70.6	Presence of G allele
*CAT*-262T (T allele)	0.63	0.54–0.72	52.9	73.5	Presence of T allele
*GSTP1* Ala114Val (T allele)	0.57	0.48–0.66	30.6	83.8	Presence of T allele
*SEPS1* G-105A (A allele)	0.66	0.57–0.75	57.6	72.1	Presence of A allele
Combination of polymorphisms (GRS)	0.74	0.67–0.81	68.2	76.5	GRS ≥ 3

## Data Availability

The original contributions presented in this study are included in the article. Further inquiries can be directed to the corresponding author.

## Abbreviations

STIs	Sexually transmitted infections
PCR	Polymerase chain reaction
Real-Time-PCR	Polymerase chain reaction in real time
CI	Confidence interval
ROS	Reactive Oxygen Species
*MTHFR*	gene encoding methylenetetrahydrofolate reductase
*MTRR*	gene encoding methionine synthase reductase
*MTR*	gene encoding methionine synthase
Me	Methyl group
SAM	S-adenosylmethionine
SNP	Single nucleotide polymorphism
